# GATE: an efficient procedure in study of pleiotropic genetic associations

**DOI:** 10.1186/s12864-017-3928-7

**Published:** 2017-07-21

**Authors:** Wei Zhang, Liu Yang, Larry L. Tang, Aiyi Liu, James L. Mills, Yuanchang Sun, Qizhai Li

**Affiliations:** 1 Key Laboratory of Systems and Control, Academy of Mathematics and Systems Science, Chinese Academy of Sciences, Beijing, China; 2 Department of Biostatistics, School of Public Health, Yale University, New Haven, CT USA; 3 College of Geoscience and Surveying Engineering, China University of Mining and Technology, Beijing, China; 4 Department of Statistics, George Mason University, Fairfax, VA USA; 5 Division of Intramural Population Health Research, Eunice Kennedy Shriver National Institute of Child Health and Human Development, National Institutes of Health, Bethesda, MD USA; 6 Department of Mathematics and Statistics, Florida International University, Miami, FL USA; 7 Rehabilitation Medicine Department, National Institutes of Health Clinical Center, Bethesda, MD USA

**Keywords:** Pleiotropic genetic associations, Principal component analysis, Power, Biomedical study

## Abstract

**Background:**

The association studies on human complex traits are admittedly propitious to identify deleterious genetic markers. Compared to single-trait analyses, multiple-trait analyses can arguably make better use of the information on both traits and markers, and thus improve statistical power of association tests prominently. Principal component analysis (PCA) is a well-known useful tool in multivariate analysis and can be applied to this task. Generally, PCA is first performed on all traits and then a certain number of top principal components (PCs) that explain most of the trait variations are selected to construct the test statistics. However, under some situations, only utilizing these top PCs would lead to a loss of important evidences from discarded PCs and thus makes the capability compromised.

**Methods:**

To overcome this drawback while keeping the advantages of using the top PCs, we propose a group accumulated test evidence (GATE) procedure. By dividing the PCs which is sorted in the descending order according to the corresponding eigenvalues into a few groups, GATE integrates the information of traits at the group level.

**Results:**

Simulation studies demonstrate the superiority of the proposed approach over several existing methods in terms of statistical power. Sometimes, the increase of power can reach 25%. These methods are further illustrated using the Heterogeneous Stock Mice data which is collected from a quantitative genome-wide association study.

**Conclusions:**

Overall, GATE provides a powerful test for pleiotropic genetic associations.

**Electronic supplementary material:**

The online version of this article (doi:10.1186/s12864-017-3928-7) contains supplementary material, which is available to authorized users.

## Background

A lot of human complex traits are highly correlated due to genetics, environmental influences and interactions among them, such as, low density lipoprotein and triglycerides, serum calcium and phosphorus, serum prostate specific antigen and prostate cancer [1–3]. Identification genetic variants that are associated with these correlated traits can help researchers understand their genetic architecture better [4]. Single nucleotide polymorphism (SNP) is an important genetic factor. A variety of SNPs have been detected to be deleterious based on the hypothesis analyses of multiple-trait-single-marker. For example, seven SNPs including rs3764261, rs4420638, rs629301, rs964184, rs1367117, rs1042034, and rs174546 are concurrently associated with four complex traits including total cholesterol, high and low density lipoprotein, and triglycerides [1, 5], and the SNP rs2476601 has been reported to be associated with five traits including rheumatoid arthritis [6], Crohn’s disease [7], systemic lupus erythematosus [8], type I diabetes [9], and Graves’ disease [10].

The joint analysis of the associations between multiple traits and a single marker is becoming popular nowadays, and many methods have been put forward in the literature [5, 11–19]. Broadly speaking, these methods can be classified into two categories: univariate analyses and multivariate analyses. The basic idea of univariate analyses is to implement the association study on one trait and one SNP firstly and then combine the obtained *p*-values with some *p*-value combination procedure to construct an omnibus test. Fisher-combined *p*-values [20] and weighted *p*-values [16] are two representative approaches of this type. Multivariate analyses mainly consist of two types of methods: model-based analyses and dimension-reduction methods. For model-based analyses, the traits are regressed on the marker or the marker is regressed on the traits simultaneously. The frequently used regression models are the mixed effect model and the proportional odds model [5, 21, 22]. Through using random effects to account for the correlation among subjects, linear mixed effect model can not only model the covariance structure caused by correlated phenotypes, but also by population structure [12, 18]. Besides, Bayesian approach is another important type of model-based approaches. PEER [23] and mvBIMBAM [15] are two Bayesian approaches which utilize the inferred hidden factor and posterior probabilities which can provide information about which phenotypes are involved in the association model. In the other hand, the canonical correlation analysis (CCA) and principal component analysis (PCA) are two common dimension-reduction approaches. Both of them have been widely applied in pleiotropic genetic association studies [17, 24, 25].

As is well known, Fisher-combined *p*-values possesses the optimal Bahadur efficiency when these *p*-values are independent [26]. However, in pleiotropic genetic studies, the test statistics are often dependent. For example, the largest value of the correlation coefficients among the traits in the Trinity Students Study analyzed below is 0.98. TATES, a typical procedure of weighted *p*-values, uses extended Simes procedure to correct for correlations among components, and might have low power when the genetic variant just affects some of the highly correlated traits. MultiPhen [5] which utilizes the proportional odds model by taking the marker as the outcome and the traits as the independent variables, may suffer from loss of power when the interested genetic marker is associated with all traits which are strongly correlated. CCA [25] is equivalent to the one-way multivariate analysis of variance analysis (MANOVA). The principal component analysis is mainly proceeded based on some top principal components (PCs) that can explain most of the total phenotypic variance of the traits used in the association studies. It will lose power if the discarded PCs are highly correlated with the traits. However, there is no widely accepted selection criterion for the optimal PCs. Furthermore, Aschard et al. [17] pointed out that the PCs that account for a small proportion of total variance can be as important as those account for a large proportion of variance in the association studies. To avoid it, they developed a multistep combined PC procedure (mCPC). For their method, the number of top PCs included in the first group is a key, which will affect the power significantly. For the selection of number of PCs, the accumulated contribution rate of 80% is recommended. As shown in the later simulation studies, using 80% sometime can lose power prominently.

In this work, we propose a procedure called GATE to test for the asscoaition between multiple traits and a single marker. GATE can be implemented using the following three steps: 1) first perform the PCA on all traits and calculate the *p*-values of the association analysis on univariate PC and a single marker one by one; 2) then divide the obtained *p*-values which are sorted by the descending order according to the correponding eigenvalues of the covariance matrix of traits into a few groups with given sizes and utilize the Fisher-combined method to combine *p*-values within and between groups; 3) let the number of *p*-values assigned in the first group vary and take the minimal value of all the quantities obatined in Step 2 as the final test statistic. To improve the computational efficiency, we propose a resampling procedure which integrates a two-layer resampling procedure to one-layer procedure to calculate the statistical significance of the test statistic. It is built based on the facts that under the null hypothesis where the genetic marker is not associated with the traits, all the *p*-values asymptotically follow the uniform distribution on [0,1] and −2 ln(*p*−value)s follow the Chi-squared distribution with 2 degrees of freedom. Simulation studies show that GATE outperforms TATES, MultiPhen and mCPC under most scenarios in terms of power. Sometime more than 25% power increase can be achieved (see Fig. 3 below). The performance of the compared methods are further illustrated using the genotypic and phenotypic data from the Trinity Students Study, a quantitative genome-wide association study.

## Methods

### The GATE

Suppose that there are *n* unrelated individuals enrolled from a source population in a genetic study. For the *i*th individual, let *y*_*ij*_ be the observation values of the *j*th trait and denote its genotype at a SNP locus by *g*_*i*_, *i*=1,2,⋯,*n*, *j*=1,2,⋯,*m*, where *m* is the number of traits of interest. Denote *Y*=(*y*_*ij*_)_*n*×*m*_ and *G*=(*g*_1_,*g*_2_,⋯,*g*_*n*_)^*τ*^. Let $\Delta =\left (\delta _{j_{1}j_{2}}\right)_{m\times m}$ be the covariance matrix of traits with $\delta _{j_{1}j_{2}}=\frac {1}{n-1}\sum \limits _{i=1}^{n}(y_{ij_{1}}-\bar y_{\cdot j_{1}})(y_{ij_{2}}-\bar y_{\cdot j_{2}})$, $\bar {y}_{\cdot j}=\frac {1}{n}\sum \limits _{i=1}^{n}y_{ij}$, *j*,*j*_1_,*j*_2_=1,2,⋯,*m*. By the singular value decomposition, *Δ* can be written as *Δ*=*Q* *Λ* *Q* ^*τ*^, where *Λ* is a diagonal matrix with diagonal elements being *λ*_1_,*λ*_2_,⋯,*λ*_*m*_ (*λ*_1_≥*λ*_2_≥⋯≥*λ*_*m*_≥0) and *Q* is an orthogonal matrix with columns being the eigenvectors. Denote *Z*=*Y* *Q*, which is called the principal component matrix whose columns correspond to all principal components. Let *z*_*j*_ be the *j*th column vector of *Z*. So the relationship between the traits and the genotype can be transformed into

$$z_{j}=\alpha_{j} + \beta_{j}G + \varepsilon_{j},~~j=1,2,\cdots,m, $$

where *ε*_*j*_ is the residual error term independently following from a normal distribution with mean of zero and unknown variance of *σ* ^2^. The null hypothesis that there is no association between the genetic variant and phenotypes becomes *H*_0_:*β*_1_=*β*_2_=⋯=*β*_*m*_=0. Denote the Wald test statistic for *β*_*j*_=0 by *T*_*j*_, *j*=1,2,⋯,*m*. Then *T*_1_,*T*_2_,⋯,*T*_*m*_ are independently and identically distributed and follow from the standard normal distribution asymptotically under the null hypothesis.

To test *H*_0_, a natural choice is the Fisher’s combined test denoted as $\text {FCT}=\sum \limits _{j=1}^{m} T_{j}^{2}$ which follows from the central Chi-squared distribution with *m* degrees of freedom (DF) asymptotically. Notice that *T*_1_,*T*_2_,⋯,*T*_*m*_ are sorted by the descending order of the eigenvalues *λ*_1_≥*λ*_2_≥⋯≥*λ*_*m*_. Aschard et al. (2014) proposed to use

$${\begin{aligned} \text{mCPC}&=-2\ln\left(1-F_{s}\left(\sum\limits_{j=1}^{s}T_{j}^{2}\right)\right)\\ & \quad -2\ln\left(1-F_{m-s}\left(\sum\limits_{j=s+1}^{m}T_{j}^{2}\right)\right), \end{aligned}} $$

where *s* is the smallest integer satisfying $\sum \limits _{j=1}^{s}\lambda _{j}\Big / \sum \limits _{j=1}^{m}\lambda _{j}\geq 0.8$, *j*=1,2,⋯,*m*, and *F*_*d*_(·) is the cumulative distribution function of the centralized chi-squared distribution with *d* DFs. As pointed out in the later simulations, using 0.8 to determine *s* is not robust and mCPC could loss power substantially. Sometimes such power loss can be more than 25% (see Fig. 3 below). In order to overcome this drawback, we suggest to divide all marginal test statistics *T*_1_,*T*_2_,⋯,*T*_*m*_ into *K* groups: $\phantom {\dot {i}\!}\{T_{1},T_{2},\cdots,T_{m_{1}}\}$, $\{T_{m_{1}+1},T_{m_{1}+2},\cdots,T_{m_{1}+m_{2}}\}$, ⋯, $\{T_{m_{1}+m_{2}+\cdots +m_{K-1}+1},T_{m_{1}+m_{2}+\cdots +m_{K-1}+2},\cdots,T_{m}\}$, where *m*_*i*_ denote the size of the *i*th groups, 0<*m*_*t*_≤*m*,*t*=1,2,⋯,*K*, and *m*_1_+*m*_2_+⋯+*m*_*K*_=*m*. For a given grouping (i.e. *m*_1_,*m*_2_,⋯,*m*_*K*_ are fixed), we can first construct a combined statistic as

$${}\begin{aligned} \xi_{m_{1}m_{2}\cdots m_{K}}&=-2\ln\left(1-F_{m_{1}}\left(\sum\limits_{j=1}^{m_{1}}T_{j}^{2}\right)\right)\\ & \quad -2\ln\left(1-F_{m_{2}}\left(\sum\limits_{j=m_{1}+1}^{m_{1}+m_{2}}T_{j}^{2}\right)\right)- \cdots\\ &\quad -2\ln\left(1-F_{m_{K}}\left(\sum\limits_{j=m_{1}+\cdots+m_{K-1}+1}^{m}T_{j}^{2}\right)\right), \end{aligned} $$

where *F*_*d*_(·) is the cumulative distribution function of the centralized Chi-squared distribution with *d* DFs and $\xi _{m_{1}m_{2}\cdots m_{K}}$ asymptotically follows from $\chi ^{2}_{2K}$ under the null hypothesis, a central Chi-squared distribution with 2*K* DFs. It should be noted that when *K*=1, although the DF of the distribution of $\xi _{m_{1}}$ is 2, the power of $\xi _{m_{1}}$ is exactly equal to that of FCT. Hence the proposed test statistic is given by

$${}{\begin{aligned} \text{GATE}=\min\limits_{K=1,2,\cdots,m-1}\left\{1-H_{K}\left(\max\limits_{m_{1}+m_{2}+\cdots+m_{K}=m}\xi_{m_{1}m_{2}\cdots m_{K}}\right)\right\}, \end{aligned}} $$

where *H*_*K*_(·) is the cumulative distribution function of $\max \limits _{m_{1}+m_{2}+\cdots +m_{K}=m}\xi _{m_{1}m_{2}\cdots m_{K}}$, 0<*m*_*t*_≤*m*,*t*=1,2,⋯,*K*. Note that when *K*=1, GATE is reduced to FCT and becomes mCPC when *K*=2,*m*_1_=*s*. Hence GATE is expected to have more broader application than FCT and mCPC.

### Significance computation

GATE is the minimal value of some correlated statistic, its exact distribution or asymptotic distribution is hard to know. To calculate the *p*-value of GATE, we propose to adopt the following resampling procedure. Since the distribution function of the statistic $\xi _{m_{1}m_{2}\cdots m_{K}}$ under each possible grouping is unknown, a two-layer resampling procedure is required. However, the two-layer resampling procedure is computation-intensive. To address it, we develop to use the following one-layer resampling procedure:

- Calculate GATE based on the observations, denote it by *η* ^(0)^. Set a large number *B*, for example *B*=10000;
- For *b* from 1 to *B*, generate *m* random variables which are *i.i.d.* from the standard normal distribution and denoted as $T_{1}^{(b)},T_{2}^{(b)}, \cdots,T_{m}^{(b)}$. Then calculate $\xi _{m_{1}m_{2}\cdots m_{K}}$ with $T_{1}^{(b)},T_{2}^{(b)}, \cdots,T_{m}^{(b)}$;
- Estimate the distribution function *H* with the $\xi _{m_{1}m_{2}\cdots m_{K}}$ obtained from Step 2 and denoted as $\hat H$;
- For *b* from 1 to *B*, using $T_{1}^{(b)},T_{2}^{(b)}, \cdots,T_{m}^{(b)}$ and $\hat H$ to calculate the GATE, denote it by *η* ^(*b*)^;
- The *p*-value of the GATE is calculated as
  $$\textit{p}-\text{value}=\frac{\#\left\{\eta^{(b)}>\eta^{(0)}:b=1,2,\cdots,B\right\}}{B}, $$
  where *#* is an operator that counts the number of the elements in a set.

We point out that when *m* is fixed, the empirical null distribution functions of *ξ* and GATE are fixed, which is free of the marker. Hence GATE can be readily to be applied to a large-scale genetic study such as genome-wide association studies.

## Results

### Simulations

#### Association models

Since the effect of a causal genetic variant on the phenotypes can be indirect and direct [27], here we consider two association models (indirect and direct association model) with indirect and direct genetic effect to generate multiple correlated phenotypes. These two models (denoted by Model 1 and Model 2) have also been used in van der Sluis et al. [16] and Aschard et al. [17]. In Model 1, the genetic markers are associated with the phenotypes through latent factors. Considering *m* correlated phenotypes, *Y*_1_,*Y*_2_,⋯,*Y*_*m*_, which depend on *L* latent variables *U*_1_,*U*_2_,⋯,*U*_*L*_ and a genetic marker *G*. Model 1 can be expressed as:

$$\left\{\begin{array}{ccc} U_{1}&=&G\beta_{1}+e_{1}\\ U_{2}&=&G\beta_{2}+e_{2}\\ \vdots\\ U_{L}&=&G\beta_{L}+e_{L} \end{array}\right. \quad \text{and} \quad \left\{\begin{array}{ccc} Y_{1}&=&U_{k_{1}}\gamma_{1}+\varepsilon_{1}\\ Y_{2}&=&U_{k_{2}}\gamma_{2}+\varepsilon_{2}\\ \vdots\\ Y_{m}&=&U_{k_{m}}\gamma_{m}+\varepsilon_{m} \end{array},\right. $$

where *k*_1_,*k*_2_,⋯,*k*_*m*_∈{1,2,⋯,*L*}, *e*_1_,*e*_2_,⋯,*e*_*L*_ and *ε*_1_,*ε*_2_,⋯,*ε*_*m*_ are independent random error terms which follow the standard normal distribution. Denote *G* as the genotype value for a biallelic SNP with the minor allele frequency being *p* (MAF=*p*) and assume that Hardy-Weinberg equilibrium holds in the general population on the SNP locus. Thus the corresponding genotype frequencies are Pr(*G*=0)=(1−*p*)^2^, Pr(*G*=1)=2*p*(1−*p*) and Pr(*G*=2)=*p* ^2^. It should be noted that in reality, the latent variables are unobservable. The correlations among phenotypes rely on the coefficients *β*=(*β*_1_,*β*_2_,⋯,*β*_*L*_)^*τ*^ and *γ*=(*γ*_1_,*γ*_2_,⋯,*γ*_*m*_)^*τ*^, which measures the strength of the association between the genetic marker and the latent variables and the association between the latent variables and the traits, respectively. The proportion of the variance of the *i*th phenotype explained by the genetic variant is $[2p(1-p)\beta _{k_{i}}^{2}\gamma _{i}^{2}]/[1+\gamma _{i}^{2}+2p(1-p)\beta _{k_{i}}^{2}\gamma _{i}^{2}]$, *i*=1,2⋯,*m*.

**Fig. 1:**
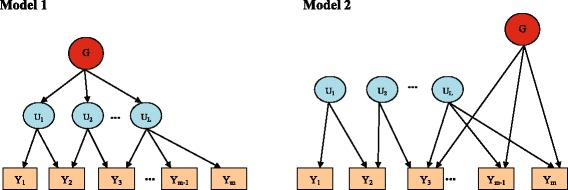
Schematic representations of two association models with indirect (Model 1) and direct (Model 2) genetic effects used to generate multiple correlated phenotypes. In Model 1, the genetic variant affects the correlated phenotypes via latent factors, while the genetic variant directly affects some single phenotypes in Model 2

For Model 2, the genetic markers are directly associated with the phenotypes and their genetic effects are independent of the latent factors. The relationships are

$${}\begin{aligned} \left\{\begin{array}{ccc} Y_{1}&=&U_{1}\gamma_{11} + U_{2}\gamma_{12} +\cdots + U_{L}\gamma_{1L}+ G\beta_{1} + \varepsilon_{1}\\ Y_{2}&=&U_{1}\gamma_{21} + U_{2}\gamma_{22} +\cdots + U_{L}\gamma_{2L}+ G\beta_{2} + \varepsilon_{2}\\ \vdots\\ Y_{m}&=&U_{1}\gamma_{m1} + U_{2}\gamma_{m2} +\cdots + U_{L}\gamma_{mL}+ G\beta_{m} + \varepsilon_{m}, \end{array}\right., \end{aligned} $$

where *U*_1_,*U*_2_,⋯,*U*_*L*_ are *L* latent variables that are independently normally distributed with mean 0 and variance 1, *G* and *ε*_1_,*ε*_2_,⋯,*ε*_*m*_ are defined as above, *γ*_*ik*_ and *β*_*i*_ are coefficients, *i*=1,2,⋯,*m*, *k*=1,2,⋯,*L*. The proportion of the variance of the *i*th phenotype explained by *G* can be calculated by $[2p(1-p)\beta _{i}^{2}]/[\sum _{k=1}^{L}\gamma _{ik}^{2}+1+2p(1-p)\beta _{i}^{2}]$, *i*=1,2⋯,*m*. These two simulated schemes are illustrated in Fig. 1.

#### Simulation settings

To compare our proposed method with the existing methods, we generate datasets from the indirect and direct association model, respectively. The detailed setups are as follows.

#### 1) Indirect association model

We set the number of latent factors to be smaller than that of phenotypes: *L*=*m*/4. Besides, we assume that every four phenotypes subject to one common latent factor and the respective effects are the same. Then Model 1 becomes *Y*_*i*_=*U*_⌈*i*/4⌉_ *γ*_*i*_+*ε*_*i*_, *U*_⌈*i*/4⌉_=*G* *β*_⌈*i*/4⌉_+*e*_⌈*i*/4⌉_, *i*=1,2,⋯,*m*, where ⌈*i*/4⌉ denote the smallest integer that is greater than *i*/4, and $r_{i_{1}}=r_{i_{2}}$ if ⌈*i*_1_/4⌉=⌈*i*_2_/4⌉, *i*_1_,*i*_2_∈{1,2,⋯,*m*}. Actually, there are *L* different values for *γ* and denote them by $\tilde {\gamma }=(\tilde {\gamma }_{1}, \tilde {\gamma }_{2},\cdots, \tilde {\gamma }_{L})^{\tau }=(\gamma _{1},\gamma _{5},\cdots,\gamma _{4k-3}, \cdots, \gamma _{m-3})^{\tau }$, *k*=1,2⋯,*L*. For a meaningful comparison, we simulate *m*=20,100 correlated traits under four patterns of correlation structures: (1) uniform low correlation; (2) uniform strong correlation; (3) a gradient of moderate to low correlations; (4) a gradient of strong to moderate correlations. Thus the derived correlation matrix under the indirect trait model is *L*-block diagonal. Denote the correlation matrix among *m* phenotypes by

$$\Delta=\left(\begin{array}{cccc} \Delta_{1} & & & \\ &\Delta_{2} & & \\ & &\ddots & \\ & & &\Delta_{L}\\ \end{array} \right)\triangleq\text{diag}(\Delta_{1},\Delta_{2},\cdots,\Delta_{L}),$$

where $\Delta _{i}=\Big (\delta _{st}^{(i)}\Big)_{\frac {m}{L}\times \frac {m}{L}}$, *i*=1,2,⋯,*L* are *m*/*L*×*m*/*L* positive definite matrices.

We specify different values for the latent variable coefficients *γ*_*i*_, *i*=1,2,⋯,*m* to ensure the non-zero elements of the correlation matrix *Δ* match the above four structures under the null hypothesis (i.e. *β*_1_=*β*_2_=⋯=*β*_*L*_=0). For uniform low and high correlation structure, we let all *γ*_*i*_,*i*=1,2⋯,*m* be equal to 0.5 and 2 which results in a uniform correlation matrix with equal correlation coefficient of 0.2 and 0.8, respectively. On the other hand, we consider a list of monotone decreasing values for *γ* to construct a gradient correlation matrix. When *m*=20, we have *L*=5 and set $\tilde {\gamma }=(1, 0.8, 0.6, 0.4, 0.2)^{\tau }$. The derived correlation matrix *Δ* belongs to the third pattern of correlation matrix and have the biggest non-zero correlation coefficient of 0.500 (moderate) and the smallest value of 0.038 (low). We set $\tilde {\gamma }=(1.5, 1.3, 1.1, 0.9, 0.7)^{\tau }$ to get the fourth pattern of correlation structure with the biggest value of 0.692 and the smallest value of 0.329 for the non-zero correlation coefficients when *m*=20. We denote the obtained four correlation structures for the indirect association model by S1, S2, S3, and S4, respectively. The detailed settings of *γ*_*i*_, *i*=1,2⋯,*m* corresponding to the above four correlation structures for *m*=20 are presented as follows:

- $\tilde {\gamma }=\left (0.5,0.5,0.5,0.5,0.5\right)^{\tau }$; $\Delta _{1}=\cdots =\Delta _{5}=\left (\begin {array}{cccc} 1 &0.2&0.2&0.2\\ 0.2& 1&0.2&0.2\\ 0.2&0.2& 1&0.2\\ 0.2&0.2&0.2&1\\ \end {array}\right)$;
- $\tilde {\gamma }=\left (2.0,2.0,2.0,2.0,2.0\right)^{\tau }$; $\Delta _{1}=\cdots =\Delta _{5}=\left (\begin {array}{cccc} 1 &0.8&0.8&0.8\\ 0.8& 1&0.8&0.8\\ 0.8&0.8& 1&0.8\\ 0.8&0.8&0.8&1\\ \end {array}\right)$;
- $\tilde {\gamma }=\left (1.0, 0.8, 0.6, 0.4, 0.2\right)^{\tau }$; $\Delta _{1}=\left (\delta _{st}^{(1)}\right)_{4\times 4}, ~\delta _{ss}^{(1)}=1,~ \delta _{st}^{(1)}=0.500 ~\text {when}~ s\neq t$; $\Delta _{2}=\left (\delta _{st}^{(2)}\right)_{4\times 4}, ~\delta _{ss}^{(2)}=1, \delta _{st}^{(2)}=0.390, ~\text {when}~ s\neq t$; $\Delta _{3}=\left (\delta _{st}^{(3)}\right)_{4\times 4}, ~\delta _{ss}^{(3)}=1, \delta _{st}^{(3)}=0.265 ~\text {when}~ s\neq t$; $\Delta _{4}=\left (\delta _{st}^{(4)}\right)_{4\times 4}, ~\delta _{ss}^{(4)}=1, \delta _{st}^{(4)}=0.138 ~\text {when}~ s\neq t$; $\Delta _{5}=\left (\delta _{st}^{(5)}\right)_{4\times 4}, ~\delta _{ss}^{(5)}=1, \delta _{st}^{(5)}=0.038 ~\text {when}~ s\neq t$;
- $\tilde {\gamma }=\left (1.5, 1.3, 1.1, 0.9, 0.7\right)^{\tau }$; $\Delta _{1}=\left (\delta _{st}^{(1)}\right)_{4\times 4}, ~\delta _{ss}^{(1)}=1, \delta _{st}^{(1)}=0.692 ~\text {when}~ s\neq t$; $\Delta _{2}=\left (\delta _{st}^{(2)}\right)_{4\times 4}, ~\delta _{ss}^{(2)}=1, \delta _{st}^{(2)}=0.628 ~\text {when}~s\neq t$; $\Delta _{3}=\left (\delta _{st}^{(3)}\right)_{4\times 4}, ~\delta _{ss}^{(3)}=1, \delta _{st}^{(3)}=0.548 ~\text {when}~s\neq t$; $\Delta _{4}=\left (\delta _{st}^{(4)}\right)_{4\times 4}, ~\delta _{ss}^{(4)}=1, \delta _{st}^{(4)}=0.448 ~\text {when}~s\neq t$; $\Delta _{5}=\left (\delta _{st}^{(5)}\right)_{4\times 4}, ~\delta _{ss}^{(5)}=1, \delta _{st}^{(5)}=0.329 ~\text {when}~s\neq t$.

The correlation matrix *Δ* is calculated under the null hypothesis (*β*_1_=*β*_2_=⋯=*β*_5_=0). Similarly, we simulated 100 correlated phenotypes with the above third and forth correlation structures through letting $\tilde {\gamma }_{i}=1-0.04(i-1)$ and $\tilde {\gamma }_{i}=1.5-0.04(i-1)$, *i*=1,2⋯,25, respectively. In addition, we provide the detailed settings of correlation structures when 100 correlated phenotypes are considered in Additional file 1 which we denote by S5, S6, S7, and S8, respectively. We specify the minor allele frequency (MAF) of the genetic variant as MAF∈{0.05,0.15,0.30,0.50}. To make the powers of all procedures comparable, 1,500 independent individuals are simulated when MAF=0.05,0.15, and 1,000 unrelated individuals are used for MAF=0.30,0.50. 1,000 simulations are conducted for the nominal significance level of 0.05.

#### 2) Direct association model

For the direct association model, the effect of latent variables is independent with that of genetic variants. Without loss of generality, we consider the structure that all the phenotypes are related to one common latent variable. Then Model 2 becomes *Y*_*i*_=*U* *γ*_*i*_+*G* *β*_*i*_+*ε*_*i*_, *i*=1,2,⋯,*m*, where *G* is the genotype vector. Similarly, we simulate *m*=20,100 correlated traits under four above patterns of correlation structures. All *γ*_*i*_, *i*=1,2⋯,*m* are specified as 0.5 and 2, respectively, for the uniform correlation matrix (denote by S9 and S10, respectively) with equal correlation coefficient of 0.2 and 0.8. In order to construct a gradient correlation matrix, we consider a list of monotone decreasing values for *γ*. When *γ*=(1.00,1.45,⋯,0.05)^*τ*^, *γ*_*i*_=1.00−0.05(*i*−1), *i*=1,2⋯,*m*−1, the resulting correlation matrix *Σ* belongs to the third pattern (denote by S11) with the values decreasing from left to right and have the biggest correlation coefficient of 0.48 (moderate) and the smallest value of 0.005 (low). We set *γ*=(1.50,1.45,⋯,0.55)^*τ*^, *γ*_*i*_=1.50−0.05(*i*−1), *i*=1,2⋯,*m*−1, to get the fourth pattern of correlation structure (denote by S12) with the biggest value of 0.68 and the smallest value of 0.25. Four patterns of correlation structures used for *m*=20 when the phenotypes are sampled from Model 2 are

- *γ*_*i*_=0.50, *i*=1,2,⋯,20; *Δ*=(*δ*_*st*_)_20×20_,*δ*_*ss*_=1, *δ*_*st*_=0.2 when *s*≠*t*;
- *γ*_*i*_=2.00, *i*=1,2,⋯,20; *Δ*=(*δ*_*st*_)_20×20_,*δ*_*ss*_=1, *δ*_*st*_=0.8 when *s*≠*t*;
- *γ*_*i*_=1.00−0.05(*i*−1),*i*=1,2,⋯,20; $\Delta =\left (\begin {array}{ccccc} 1&0.480&\cdots &0.070&0.035\\ 0.480&1&\cdots &0.680&0.034\\ \vdots &\vdots &\ddots &&\\ 0.070&0.068&\cdots &1&0.005\\ 0.035&0.034&\cdots &0.005&1\\ \end {array}\right)_{20\times 20};$
- *γ*_*i*_=1.50−0.05(*i*−1),*i*=1,2,⋯,20; $\Delta =\left (\begin {array}{ccccc} 1&0.680&\cdots &0.420&0.400\\ 0.680&1&\cdots &0.420&0.390\\ \vdots &\vdots &\ddots &&\\ 0.420&0.420&\cdots &1&0.250\\ 0.400&0.390&\cdots &0.250&1\\ \end {array}\right)_{20\times 20}.$

Likewise, these correlation matrices are calculated under the null hypothesis (*β*_1_=*β*_2_=⋯=*β*_20_=0). And we also summarize the settings of four corresponding correlation structures (denote by S13, S14, S15, and S16) for *m*=100 which is provided detailedly in the Additional file 1. In the following simulation, MAF∈{0.05,0.15,0.30,0.50} are considered.

#### Selection of *k*

The selection of *K* in GATE is a key since large *K* leads to extensive computations and small *K* may result in not grasping the information thoroughly. We suggest selecting *K*=2 for the proposed GATE procedure in practice. From the view of “pseudo degree of freedom”, when *K*=1, the statistic $\xi _{m_{1}m_{2}\cdots m_{K}}$ which is used in the construction of the GATE statistic might possess *m* degrees of freedom or so, while when *K*≥2, the corresponding DF becomes 2*K*. Thus, when the number of traits that need to be analysed is enough large (*m*>4), dividing all single Wald test statistics *T*_1_,*T*_2_,⋯,*T*_*m*_ into 2 groups will lead to the smallest DF. Hence, we deduce that the GATE with *K*=2 will have better power performance than the other selections of *K*. Furthermore, we conduct some simulation studies to explore the performances of GATE under different selections of *K*. The simulations results are summarized in the Additional file 1 which coincidentally demonstrates our deduction. Therefore, in the following simulation, we compare the GATE with only considering *K*∈{1,2} to other existing methods.

#### Performance comparison to other methods

In order to test the performance of the proposed GATE aprroach, four existing methods including TATES [16], MultiPhen [5], MANOVA, and mCPC [17] are compared.

#### 1) Indirect association model

Firstly, we assume the correlated phenotypes are sampled from the indirect association model and explore the performances of the above five tests.

#### Type I error rate

Table 1 summarizes the empirical type I error rates of these five methods under the nominal significance level of 0.05 when the correlated phenotypes are simulated from Model 1. When *m*=20, all of the five tests can control the type I error correctly with their empirical values being close to the nominal significance level. For example, when MAF=0.15,*m*=20, the empirical type I error rates of TATES, MANOVA, MultiPhen, mCPC, and GATE for the phenotypes with the correlation matrix of S3 are 0.051, 0.051, 0.049, 0.051, and 0.052, respectively. However, when the number of simulated phenotypes is large (*m*=100), MultiPhen always has inflated type I error rates. For instance, when the phenotypes are generated from the indirect association model with the correlation matrix of S5, the empirical type I errors of MultiPhen for MAF=0.05,0.15,0.30,0.50 are 0.111, 0.087, 0.096, and 0.114, respectively. So we exclude it in the following comparisons of power for 100 phenotypes.

**Table 1:** The empirical type I errors of TATES, MANOVA, MultiPhen, mCPC, and GATE when the correlated phenotypes are sampled from indirect association model

	Scenario	MAF	TATES	MANOVA	MultiPhen	mCPC	GATE
*m*=20	S1	0.05	0.046	0.044	0.048	0.046	0.043
		0.15	0.048	0.045	0.043	0.044	0.041
		0.30	0.058	0.054	0.064	0.052	0.053
		0.50	0.047	0.047	0.058	0.050	0.045
	S2	0.05	0.062	0.053	0.049	0.043	0.053
		0.15	0.059	0.058	0.060	0.057	0.054
		0.30	0.061	0.046	0.047	0.047	0.047
		0.50	0.054	0.053	0.054	0.055	0.059
	S3	0.05	0.053	0.047	0.047	0.041	0.043
		0.15	0.051	0.051	0.049	0.051	0.052
		0.30	0.064	0.053	0.057	0.061	0.065
		0.50	0.050	0.061	0.065	0.066	0.062
	S4	0.05	0.062	0.042	0.047	0.046	0.042
		0.15	0.051	0.045	0.045	0.049	0.045
		0.30	0.045	0.042	0.045	0.047	0.047
		0.50	0.052	0.044	0.049	0.060	0.057
*m*=100	S5	0.05	0.055	0.059	0.111	0.057	0.059
		0.15	0.053	0.049	0.087	0.046	0.053
		0.30	0.056	0.037	0.096	0.030	0.032
		0.50	0.060	0.051	0.114	0.038	0.051
	S6	0.05	0.058	0.049	0.103	0.046	0.051
		0.15	0.056	0.056	0.093	0.063	0.058
		0.30	0.062	0.043	0.098	0.040	0.049
		0.50	0.076	0.046	0.116	0.037	0.050
	S7	0.05	0.057	0.052	0.104	0.051	0.048
		0.15	0.045	0.045	0.086	0.058	0.056
		0.30	0.052	0.045	0.103	0.040	0.041
		0.50	0.049	0.062	0.114	0.054	0.053
	S8	0.05	0.066	0.063	0.113	0.063	0.062
		0.15	0.063	0.046	0.085	0.051	0.055
		0.30	0.039	0.052	0.116	0.041	0.038
		0.50	0.066	0.071	0.126	0.066	0.054

The number of correlated phenotypes is 20 and 100. Scenario S1-S4 correspond to four correlation structures for m=20 and Scenario S5-S8 are for m=100. For each scenario, four MAFs including 0.05, 0.15, 0.30, and 0.50 are considered. The nominal significance level is 0.05 and 1000 simulations are conducted

#### Power

Next, we compare the powers of the TATES, MANOVA, MultiPhen, mCPC, and GATE under the nominal significance level of 0.05. Under each scheme of the correlation structures, 5 levels of association including *λ*=20*%*,40*%*,60*%*,80*%*,100*%* of the phenotypes that are associated with the genotype are considered. Denote the number of the associated traits by *k* (=*λ* *m*). Without loss of generality, we assume that the first *k* phenotypes are associated with *G*. Besides this, we consider the scenarios that the phenotypes are randomly selected to be associated with the genotype and the corresponding results are presented in Additional file 1.

**Fig. 2:**
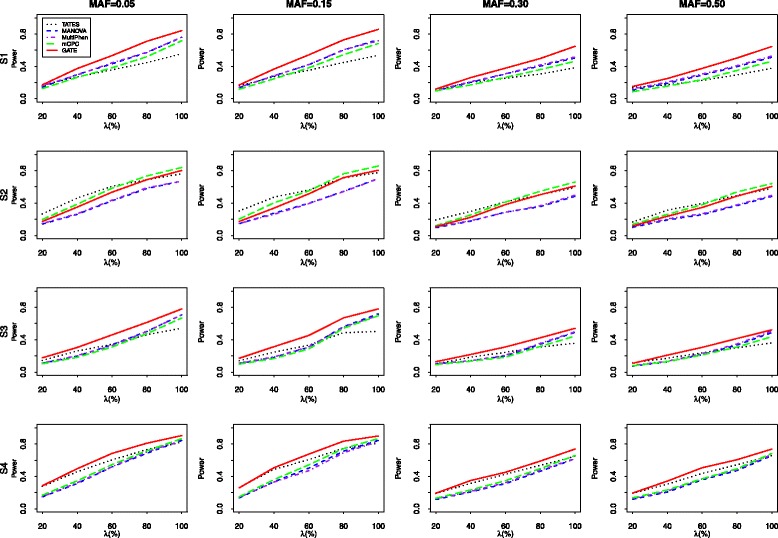
The empirical power of five tests for 20 correlated phenotypes sampled from Model 1 with correlation structure S1-S4. 1000 simulation replicates are conducted under the nominal significant level of 0.05

Figure 2 reports the power results for 20 correlated phenotypes which are generated from Model 1 with the correlation structures of S1, S2, S3, and S4, respectively. To make the power comparable, we set the proportions of the variance of the associated phenotypes explained by the genetic variant under the four configurations (S1, S2, S3, S4) are 0.1%, 0.2%, 0.1%, and 0.2%, respectively. In most cases, our proposed test is more powerful than the other methods except when the correlations among associated phenotypes are uniformly strong (S2). Sometimes the power increase of TATES compared to the other four approaches can reach 13%. For example, when MAF=0.15,*n*=1,500,*λ*=60*%* and *Σ* belongs to S3, the empirical powers of TATES, MANOVA, MultiPhen, mCPC, and GATE are 0.324, 0.309, 0.312, 0.286, and 0.453, respectively. GATE is sightly less powerful than mCPC and TATES when the non-zero correlation coefficients are uniformly equal to 0.8 (S2) and the gap between them narrows as the proportion of associated phenotypes increases. For example, under the correlation matrix of S2 and MAF=0.15,*n*=1,500, the empirical powers of TATES for *λ* = 20%, 40%, 60%, 80%, 100% are 0.166, 0.313, 0.396, 0.495, and 0.580, respectively, and those of GATE are 0.118, 0.238, 0.346, 0.486, and 0.605. We also find that MANOVA and MultiPhen usually have similar performance when the number of phenotypes are not too large.

**Fig. 3:**
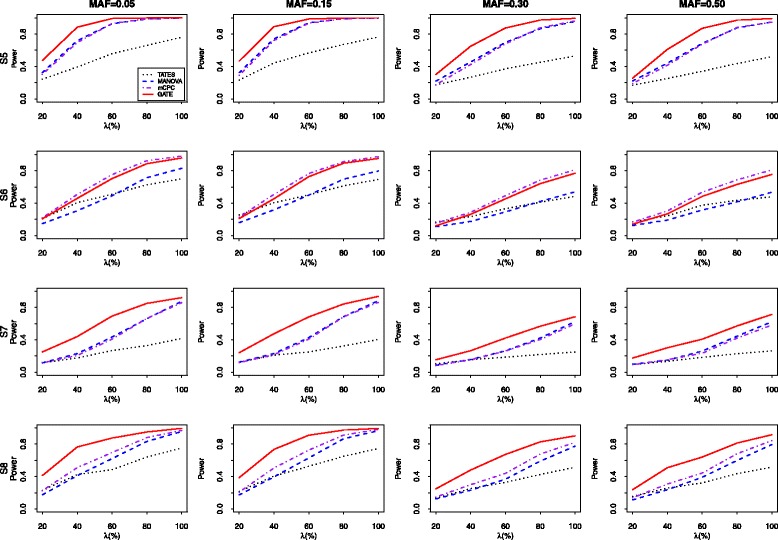
The empirical power of five tests for 100 correlated phenotypes sampled from Model 1 with correlation structure S5-S8. 1000 simulation replicates are conducted under the nominal significant level of 0.05

The empirical powers of four test (excluding MultiPhen) for 100 phenotypes which are simulated from the indirect trait model with the correlation schemes of S5, S6, S7, and S8 are summarized in Fig. 3. The proportions of the variance of the associated phenotypes explained by the genetic variant under four patterns of correlation structures are 0.1%, 0.1%, 0.05%, and 0.1%, respectively. The results for *m*=100 are similar to those for *m*=20. In most scenarios, GATE performs better than the other three methods. From Fig. 3, we can find that the superiority of GATE over the other three methods is more evident when the number of the analysed traits is large. Sometimes, the power increase of the proposed GATE can reach 25%. For instance, when MAF=0.15,*n*=1,500,*λ*=60*%* under the correlation matrix of S7, the powers of TATES, MANOVA, mCPC, and GATE are 0.251, 0.428, 0.409, and 0.683, respectively. When the non-zero correlation coefficients are uniformly large (S6), the mCPC performs slightly better than the GATE. This occurs because when the correlations among different phenotypes are strong and a relatively large number of analysed traits are analyzed, the test $\xi _{m_{1}}$ which is included in the construction of GATE would have loss of power substantially. However, under the other correlation structures (S5, S7, and S8), the powers of the GATE are always higher than those of mCPC. In some cases, GATE can have 28% increase of power comparing to mCPC. For instances, when MAF=0.05,*n*=1,500,*λ*=60*%* under the correlation matrix of S7, the powers of mCPC and GATE are 0.408 and 0.693, respectively.

#### 2) Direct association model

Next, we assess the performance of the proposed test compared with those of the other four tests when the correlated phenotypes are sampled from the direct association model.

#### Type I error rate

In this section, we compare the type I error rates of TATES, MANOVA, MultiPhen, mCPC, and GATE when multiple phenotypes are generated from direct association model. Table 2 reports the results of type I error rate for 20 and 100 correlated phenotypes under the nominal significance level of 0.05, respectively. It shows that when *m*=20, all five tests can control the type I error rates correctly because their empirical type I error rates are close to the nominal level. For example, when MAF=0.30, the type I error rates of TATES, MANOVA, MultiPhen, mCPC, and GATE under the correlation structure of S11 are 0.049, 0.054, 0.055, 0.052, and 0.054, respectively. Similarly, when the number of simulated phenotypes is large (*m*=100), MultiPhen has inflated type I error rates, while the other four tests maintain correct type I error rates. For instance, when *m*=100, the type I error rates of MultiPhen under the correlation structure of S15 for MAF=0.05,0.15,0.30, and 0.50 are 0.106, 0.102, 0.118, and 0.103, respectively.

**Table 2:** The empirical type I errors of TATES, MANOVA, MultiPhen, mCPC, and GATE when the correlated phenotypes are sampled from direct association model

	Scenario	MAF	TATES	MANOVA	MultiPhen	mCPC	GATE
*m*=20	S9	0.05	0.045	0.048	0.050	0.045	0.047
		0.15	0.054	0.049	0.058	0.050	0.048
		0.30	0.051	0.047	0.054	0.043	0.045
		0.50	0.050	0.054	0.056	0.053	0.052
	S10	0.05	0.037	0.054	0.059	0.048	0.052
		0.15	0.035	0.055	0.050	0.046	0.045
		0.30	0.038	0.049	0.055	0.052	0.043
		0.50	0.031	0.043	0.049	0.047	0.047
	S11	0.05	0.041	0.050	0.050	0.052	0.050
		0.15	0.049	0.047	0.054	0.048	0.053
		0.30	0.049	0.054	0.055	0.052	0.054
		0.50	0.060	0.053	0.060	0.052	0.055
	S12	0.05	0.042	0.051	0.057	0.062	0.064
		0.15	0.040	0.047	0.045	0.044	0.048
		0.30	0.041	0.043	0.048	0.045	0.046
		0.50	0.045	0.048	0.052	0.048	0.051
*m*=100	S13	0.05	0.049	0.044	0.086	0.042	0.044
		0.15	0.060	0.047	0.089	0.046	0.040
		0.30	0.060	0.063	0.119	0.052	0.056
		0.50	0.053	0.041	0.094	0.039	0.051
	S14	0.05	0.031	0.053	0.100	0.059	0.048
		0.15	0.024	0.059	0.092	0.046	0.054
		0.30	0.030	0.051	0.114	0.051	0.058
		0.50	0.030	0.050	0.122	0.046	0.042
	S15	0.05	0.058	0.055	0.106	0.056	0.050
		0.15	0.047	0.051	0.102	0.059	0.043
		0.30	0.042	0.066	0.118	0.061	0.051
		0.50	0.048	0.039	0.103	0.045	0.043
	S16	0.05	0.036	0.055	0.099	0.059	0.053
		0.15	0.041	0.050	0.087	0.055	0.049
		0.30	0.041	0.047	0.110	0.057	0.065
		0.50	0.042	0.065	0.130	0.059	0.052

The number of correlated phenotypes is 20 and 100. Scenario S9-S12 correspond to four correlation structures for m=20 and Scenario S13-S16 are for m=100. For each scenario, four MAFs including 0.05, 0.15, 0.30, and 0.50 are considered. The nominal significance level is 0.05 and 1000 simulation replicates are conducted

#### Power

Next, we compare the power of the five tests when multiple phenotypes are simulated from Model 2. Under each scheme of the correlation structures, 5 levels of association: *λ*=20*%*,40*%*,60*%*,80*%*,100*%* of the phenotypes that are associated with the genetic variant are considered and the number of the associated traits is *k*. Here we report the results under the scenario where the first *k* phenotypes are associated with the genetic variant. Additional empirical power results for the cases that the associated phenotypes are randomly selected with equal probability are available in Additional file 1.

**Fig. 4:**
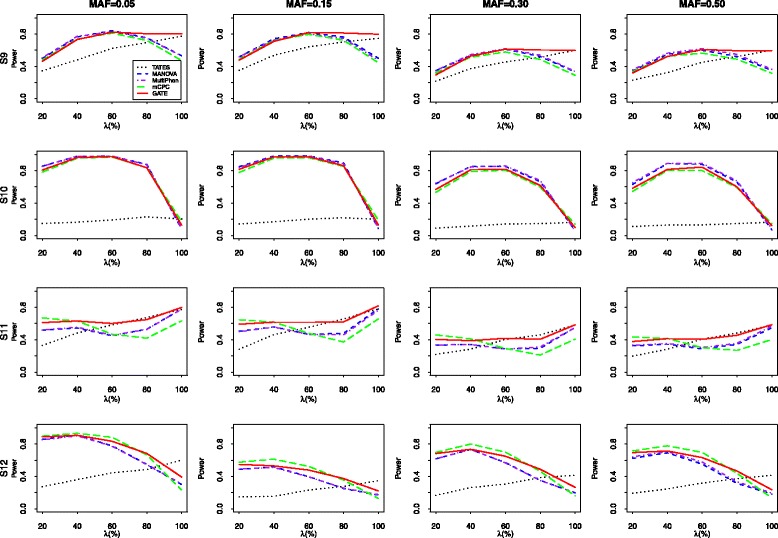
The empirical power of five tests for 20 correlated phenotypes sampled from Model 2 with correlation structure S9-S12. 1000 simulation replicates are conducted under the nominal significant level of 0.05

**Fig. 5:**
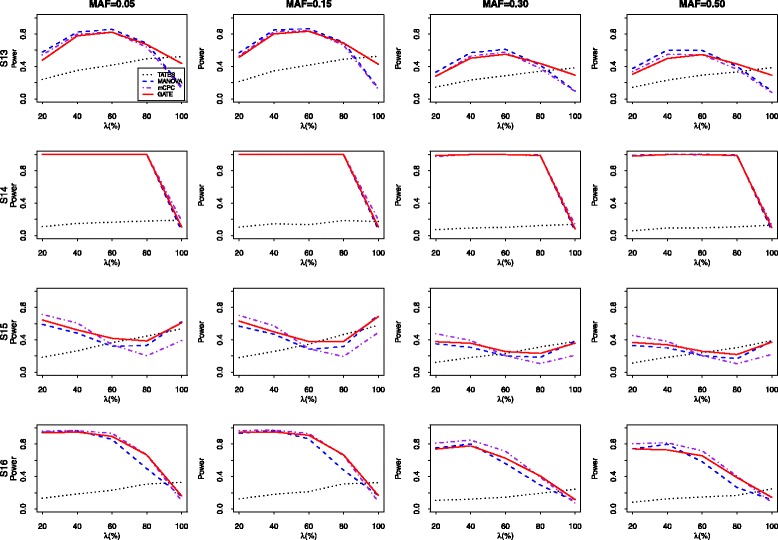
The empirical power of five tests for 100 correlated phenotypes sampled from Model 2 with correlation structure S13-S16. 1000 replicates are conducted under the nominal significant level of 0.05

Figure 4 presents the power results of all five tests for 20 correlated phenotypes simulated from Model 2 with S9, S10, S11 and S12, respectively. To make the power results comparable, we set the proportions of the variance of the associated phenotypes explained by the genetic variant under the four configurations (S9-S12) are 0.2%, 0.1%, 0.2%, and 0.2%, respectively. When *m*=20, we find that MANOVA and MultiPhen usually have similar performances. For example, when MAF=0.30 and the correlation structure is S9, the powers of MANOVA and MultiPhen for *λ*=20*%*,40*%*,60*%*,80*%*, and 100*%* are (0.348, 0.350), (0.535, 0.546), (0.612, 0.618), (0.523, 0.539), and (0.333, 0.338), respectively. When the correlations among different phenotypes are equal (S9 and S10) and the number of the associated phenotypes are relatively small (*λ*<60*%*), MANOVA and MultiPhen are two most powerful tests and they have similar power performances. In other situations, GATE performs better than MANOVA and MultiPhen. For example, under the configuration of S9 and MAF=0.15, the powers of the MANOVA, MultiPhen and GATE tests for *λ*=40*%* are 0.742,0.726 and 0.710, and their corresponding powers for *λ*=100*%* are 0.501, 0.475 and 0.798, respectively. When the correlations among different phenotypes are nonuniform (S11 and S12), the GATE performs better than the other four methods in most cases. When *λ* is relatively small, mCPC outperform slightly than GATE. However, when *λ* becomes large, the powers of the GATE exceed those of mCPC significantly. And in some cases, the power increase can reach 25%. For example, when the correlation structure is S11 and MAF=0.15, the powers of mCPC and GATE for *λ*=80*%* are 0.372 and 0.621, respectively. Moreover, when there exist strong correlations among phenotypes (S10), TATES suffers significant loss of power. For example, when MAF=0.05,*n*=1,500, and the rate of associated phenotypes with S10 is 60%, the powers of the TATES, MANOVA, MultiPhen, mCPC, and GATE are 0.192, 0.980, 0.981, 0.969, and 0.975, respectively. Hence, the proposed GATE is the most robust test against different levels of pleiotropy and strengthes of correlation. Figure 5 shows the power results of four tests including TATES, MANOVA, mCPC, and GATE for 100 simulated phenotypes from Model 2. The proportions of the variance of the associated phenotypes explained by the genetic variant under four configurations (S13, S14, S15, S16) are all set to be 0.1%. The performances of all compared approaches are similar to those under *m*=20.

### Applications to heterogeneous stock mice data

The mouse is an important model organism which can provide information on gene functions in mammals. Its use has been proved to be a powerful approach to understanding the genetic architecture of human disease and fundamental mammalian biology [28]. To further explore the performance of the proposed method on the test for pleiotropic genetic effects, we apply it to the analysis of the Heterogeneous Stock Mice data, which is downloaded from http://mus.well.ox.ac.uk/. Originally, 101 phenotypes including models of human disease (such as, asthma, type 2 diabetes mellitus, obesity, anxiety), immunological, biochemical and hematological phenotypes, and others, are collected [29]. These 101 phenotypes belong to 19 categories and a full description of them is available in Solberg et al. [30]. Before the analysis, we remove the phenotypes with the proportion of missing values being large than 0.01, so that 52 phenotypes (listed in Additional file 1) are left. The remaining phenotyps are correlated with each other and the largest correlation coefficient is 0.979, which happens between two hematological phenotypes: “Haem Haemoglobin” and “Haem Haematocrit”. In addition, we exclude the subjects with missing oberved phenotype values and thus a total of 588 mice are obtained. There are totally 302 SNPs on chromosome 19. After removing the SNPs with the proportions of missing genotype value large than 15% and MAF being smaller than 0.05, 250 SNPs are finally analyzed.

We use TATES, MANOVA, MultiPhen, mCPC, and GATE to test the association between the SNPs on chromosome 19 and all 52 phenotypes. 1,000,000 resamplings are conducted to calculate of the *p*-value of GATE. Under the nominal significance level of 0.05, the adjusted significance level for a single test is 0.05/250= 2×10^−4^ from the Bonferroni correction for multiplicity. On a whole, the number of identified SNPs that are associated with all the 52 phenotypes by GATE is more than those by the other four methods. Among 250 SNPs, there are 125 SNPs are detected by GATE, while 80, 114, 118, and 116 SNPs are detected by TATES, MANOVA, MultiPhen, and mCPC, respectively. Among the 125 SNPs, there are 7 SNPs that are only detected to be significantly associated with all the analyzed phenotypes by the proposed methods. The *p*-values of these SNPs for five methods are presented in Table 3. For each SNP, the *p*-value of GATE is always the smallest and smaller than the adjusted significance level 2×10^−4^. For example, for rs13483499, the *p*-value of GATE is 6.30×10^−5^ which is smaller than those of TATES (3.27×10^−3^), MANOVA (1.79×10^−4^), MultiPhen (3.34×10^−4^) and mCPC (1.63×10^−4^). Besides, some of the identified SNPs have been found to have implications on the analyzed phenotypes in the literature. For example, Valdar et al. [29] reported that the SNP rs13459157 is associated with the phenotype “OFT Activity and defecation”, which is among the 52 phenotypes and the SNP rs6259521 has an association with the phenotype “Pleth Enhanced pause (baseline)”.

**Table 3:** *P*-values of the selected 7 SNPs on mouse chromosome 19 for the association tests with 52 phenotypes using the TATES, MANOVA, MultiPhen, mCPC, and GATE methods

snpid	TATES	MANOVA	MultiPhen	mCPC	GATE
rs13483499	3.27 ×10^−3^	1.79 ×10^−4^	3.34 ×10^−4^	1.63 ×10^−4^	6.30 ×10^−5^
rs13459157	4.05 ×10^−3^	2.36 ×10^−4^	4.20 ×10^−4^	2.17 ×10^−4^	8.80 ×10^−5^
rs13483502	4.23 ×10^−2^	1.01 ×10^−3^	2.22 ×10^−4^	9.97 ×10^−4^	1.67 ×10^−4^
rs6259521	3.00 ×10^−3^	1.11 ×10^−2^	4.63 ×10^−3^	6.41 ×10^−4^	1.73 ×10^−4^
rs13483579	1.19 ×10^−3^	6.15 ×10^−2^	6.17 ×10^−2^	5.44 ×10^−3^	1.74 ×10^−4^
rs13483598	4.53 ×10^−3^	3.16 ×10^−3^	8.07 ×10^−4^	2.85 ×10^−3^	1.36 ×10^−4^
rs13483601	3.82 ×10^−3^	2.82 ×10^−3^	7.45 ×10^−4^	1.84 ×10^−3^	8.50 ×10^−5^

“snpid” is the ID of the selected SNPs

## Discussion

The genetic variants play fundamental roles in studies of human complex diseases. The elucidation of genetic risk factors could provide an insightful understanding on the occurrence of the diseases and then make the targeted therapy feasible. As the genome-wide association studies move forward, the association between multiple traits and a single SNP is becoming a hot pot nowadays. Intuitively, multiple-traits-single-SNP analysis (MTSS) is more powerful in identifying deleterious SNP compared to single-marker test on one trait. In this paper, we have presented GATE, a new procedure to do MTSS. The false positive rate of GATE is controlled correctly for various MAFs and different correlation structures for the traits since the computation of the significance of GATE is based on the resampling procedure. Extensive simulations including the direct association model and indirect association model show that GATE outperforms the existing procedures when the association model is indirect and the relationship is not consistently strong, and is more robust under other situations. In other words, GATE is an efficient multivariate analysis procedure to conduct association studies between genotypes and phenotypes since the potential genetic architecture is generally unknown beforehand.

We provide a resampling procedure to calculate the significance of GATE. The key of such resampling procedure is to generate i.i.d. observations from the standard normal distribution. This procedure is very user-friendly and can be implemented in any statistical and numerical softwares such as R, SAS, Matlab, and others. In principle, a two-layer resampling procedure should be employed to obtain the *p*-value of GATE. Here we adopt a one-layer resampling procedure, where the cumulative distribution function (*H*) of the inner statistic was estimated at the beginning, and then use the estimated distribution function of *H* and the same samples to obtain the empirical significance of GATE. This procedure can efficiently reduce the computational cost and make GATE feasible to a large-scale genetic study. Since a large number of replications in the one-layer resampling procedure won’t result in high computation cost, we recommend using B=10000 or larger to ensure the stability of the calculated GATE s *p*-values. On the other hand, we can use the generalized Gamma distribution (GGD) [20] to approximate the distribution of −2 ln(GATE). The 95%, 99%, 99.9%, 99.99%, 99.999% quantiles using the fitted GDD and the empirical values of −2 ln(GATE) based on 1,000,000 resamplings are given in Additional file 1: Table S5. They match very well. So in order to reduce the computational intensity, we can consider using the fitted GDD method to obtain the *p*-values of GATE. The proposed procedure has been coded in R verion 3.3.3 and is available at http://www.statsci.amss.ac.cn/yjscy/yjy/lqz/201510/t20151027_313273.html.

PCA is an important tool in multivariate analysis. In PCA, a crucial issue is how to select PCs. A standard selection criterion is using the cumulative contribution rate that indicates a few top PCs can be chosen. As pointed out by [31] and [17], only using some top PCs might miss some important PCs that are with low contribution rate, but are highly correlated with the outcome. FCT that combines all PCs can be an alternative approach. However, it loses power substantially when the number of true signals is large. To overcome this drawback, Aschard [17] proposed a mCPC procedure. By dividing the marginal test statistics for each PC into two groups and combining the tests among groups, the DF can be reduced, especially when the signals are very sparse. However, for the correlation structure among multiple phenotypes and association strength between genotype and phenotype are unknown beforehand, using a fixed grouping technique is not enough robust. GATE makes a bridge between FCT and mCPC. To some extent, GATE can be regarded as an extension of FCT and mCPC since it is exactly equal to FCT when the group number is one and takes mCPC as one of components. GATE is constructed from a large family of test statistics containing FCT and mCPC. Overall, GATE is more robust than FCT and mCPC. The simulation results also demonstrate it.

GATE is also an extension of TATES who uses the minimum of weighted *p*-value as the test statistic. Basically, TATES can be viewed as a function of *p*-values. The function is the combination of linear operator and minimum operator. For GATE, it utilizes the cumulative distribution function, log, quadratic-form and summation functions. These four functions are commonly used in constructing the test statistics in hypothesis testing, which is expected to integrate the information over a wide range of scenarios than other functions. The simulations show that GATE is more robust than TATES under most of the considered scenarios.

Covariates or confounding factors including the gender, age, environment factors can be of great importance in assessing the associations between the genetic variants and complex traits. Adjusting for covariates in genetic association studies have two motivations: one is correcting for the bias of the genetic effect estimates, and another is improving statistical power. For example, the hidden population structure can not be ignored in population genetic association studies and a failure to consider the population stratification might lead to many false positive findings. So it is a routine for researchers to correct for the population stratification in the genome wide association studies. Fortunately, the proposed GATE procedure can be directly applied to multiple traits association studies with covariates by adding the covariates in the association studies of the single PC and genetic variant.

## Conclusions

GATE is an efficient and robust procedure for association studies between multiple traits and a single SNP, which holds the key to understanding the genetic architecture of complex diseases. GATE is implemented based on the principal components (PCs) of multiple correlated traits. In contrast with the traditional PCA, GATE utilizes all obtained PCs instead of some selected PCs. This is because that low variances may possess important evidences of association and combines them in a group manner. Extensive numerical analyses show the superiority of the proposed approach over several existing methods in terms of statistical power.

## Additional file

Additional file 1 GATE: an efficient procedure in study of pleiotropic genetic associations. (PDF 317 kb)

## Abbreviations

CCA: Canonical correlation analysis
DF: Degrees of freedom
GATE: Group-accumulated test evidence
GGD: Generalized Gamma distribution MANOVA: Multivariate analysis of variance analysis
mCPC: Multistep combined PC procedure
MAF: Minor allele frequency
MTSS: Multiple-traits-single-SNP analysis
PCA: Principal component analysis
PCs: Principal components
SNP: Single nucleotide polymorphism
