# Supplementary material for: GATE: an efficient procedure in study of pleiotropic genetic associations
Source: BMC Genomics. 2017 Jul 21;18:552. doi: 10.1186/s12864-017-3928-7 (PMC5521155; doi:10.1186/s12864-017-3928-7)
Supplement: Additional file 1 — GATE: an efficient procedure in study of pleiotropic genetic associations. (PDF 317 kb) [file 12864_2017_3928_MOESM1_ESM.pdf]

Additional file for

GATE: an efficient procedure in study of pleiotropic genetic associations

Wei Zhang, Liu Yang, Larry L. Tang, Aiyi Liu, James L. Mills, Yuanchang Sun and Qizhai Li

Contents

1. Additional settings of correlation structure used in the indirect association model when $m = 100$.
2. Additional settings of correlation structure used in the direct association model when $m = 100$.
3. Simulation results for the selection of k.
4. Simulation results when the associated phenotypes are selected with equal probability from indirect association model.
5. Simulation results when the associated phenotypes are selected with equal probability from direct association model.
6. Simulation results for $m = 10$.
7. Simulation results for $m = 50$.
8. The estimation of density function of the GATE considering $k \in \{1, 2\}$.
9. The analyzed mice phenotype information.

1. Additional settings of correlation structure used in the indirect association model when $m = 100$

Table S1. Four correlation structures used in the Indirect trait model when $m = 100$. The correlation matrix Δ is calculated under the null hypothesis ($\beta_1 = \beta_2 = \cdots = \beta_{25} = 0$). Assume that $\gamma_i = \gamma_j$ if $\lceil i/4 \rceil = \lceil j/4 \rceil$, so there are $L = 25$ different values for γ_i, $i = 1, 2 \cdots, m$, which we denote as $\tilde{\gamma} = (\tilde{\gamma}_1, \tilde{\gamma}_2, \cdots, \tilde{\gamma}_{25})^\tau$.

	$\tilde{\gamma} = (\tilde{\gamma}_1, \tilde{\gamma}_2, \cdots, \tilde{\gamma}_{25})^\tau$	$\Delta = \text{diag}(\Delta_1, \Delta_2, \cdots, \Delta_{25})$ (under H_0)
S5	$\tilde{\gamma}_i = 0.50,\ i = 1, 2, \cdots, 25$	$\Delta_1 = \cdots = \Delta_{25} = \begin{pmatrix} 1 & 0.2 & 0.2 & 0.2 \\ 0.2 & 1 & 0.2 & 0.2 \\ 0.2 & 0.2 & 1 & 0.2 \\ 0.2 & 0.2 & 0.2 & 1 \end{pmatrix}$
S6	$\tilde{\gamma}_i = 2.00,\ i = 1, 2, \cdots, 25$	$\Delta_1 = \cdots = \Delta_{25} = \begin{pmatrix} 1 & 0.8 & 0.8 & 0.8 \\ 0.8 & 1 & 0.8 & 0.8 \\ 0.8 & 0.8 & 1 & 0.8 \\ 0.8 & 0.8 & 0.8 & 1 \end{pmatrix}$
S7	$\tilde{\gamma}_i = 1 - 0.04(i-1), i = 1, 2, \cdots, 25$	$\Delta_1 = (\delta_{st}^{(1)})_{4\times 4},\ \delta_{ss}^{(1)} = 1, \delta_{st}^{(1)} = 0.500,$ when $s \neq t$;<br>$\Delta_2 = (\delta_{st}^{(2)})_{4\times 4},\ \delta_{ss}^{(2)} = 1, \delta_{st}^{(2)} = 0.480,$ when $s \neq t$;<br>$\vdots$<br>$\Delta_{24} = (\delta_{st}^{(24)})_{4\times 4},\ \delta_{ss}^{(24)} = 1, \delta_{st}^{(24)} = 0.006,$ when $s \neq t$;<br>$\Delta_{25} = (\delta_{st}^{(25)})_{4\times 4},\ \delta_{ss}^{(25)} = 1, \delta_{st}^{(25)} = 0.002,$ when $s \neq t$.
S8	$\tilde{\gamma}_i = 1.50 - 0.04(i-1), i = 1, 2, \cdots, 25$	$\Delta_1 = (\delta_{st}^{(1)})_{4\times 4},\ \delta_{ss}^{(1)} = 1, \delta_{st}^{(1)} = 0.692,$ when $s \neq t$;<br>$\Delta_2 = (\delta_{st}^{(2)})_{4\times 4},\ \delta_{ss}^{(2)} = 1, \delta_{st}^{(2)} = 0.681,$ when $s \neq t$;<br>$\vdots$<br>$\Delta_{24} = (\delta_{st}^{(24)})_{4\times 4},\ \delta_{ss}^{(24)} = 1, \delta_{st}^{(24)} = 0.252,$ when $s \neq t$;<br>$\Delta_{25} = (\delta_{st}^{(25)})_{4\times 4},\ \delta_{ss}^{(25)} = 1, \delta_{st}^{(25)} = 0.226,$ when $s \neq t$.

2. Additional settings of correlation structure used in the direct association model when $m = 100$

Table S2. Four correlation structures used in the Model 2 when $m = 100$. The correlation matrix Δ is calculated under the null hypothesis ($\beta_1 = \beta_2 = \cdots = \beta_{100} = 0$).

	$\gamma = (\gamma_1, \gamma_2, \cdots, \gamma_i, \cdots, \gamma_{100})^\tau$	Δ (under H_0)
S13	$\gamma_i = 0.50,\ i = 1, 2, \cdots, 100$	$\begin{pmatrix} 1 & 0.2 & \cdots & 0.2 \\ 0.2 & 1 & \cdots & 0.2 \\ \vdots & \vdots & \ddots & \\ 0.2 & 0.2 & \cdots & 1 \end{pmatrix}_{100\times100}$
S14	$\gamma_i = 2.00,\ i = 1, 2, \cdots, 100$	$\begin{pmatrix} 1 & 0.8 & \cdots & 0.8 \\ 0.8 & 1 & \cdots & 0.8 \\ \vdots & \vdots & \ddots & \\ 0.8 & 0.8 & \cdots & 1 \end{pmatrix}_{100\times100}$
S15	$\gamma_i = 1.00 - 0.01(i-1), i = 1, 2, \cdots, 100$	$\begin{pmatrix} 1 & 0.497 & \cdots & 0.014 & 0.007 \\ 0.497 & 1 & \cdots & 0.014 & 0.007 \\ \vdots & \vdots & \ddots & & \\ 0.014 & 0.014 & \cdots & 1 & 0.0002 \\ 0.007 & 0.007 & \cdots & 0.005 & 1 \end{pmatrix}_{100\times100}$
S16	$\gamma_i = 1.50 - 0.01(i-1), i = 1, 2, \cdots, 100$	$\begin{pmatrix} 1 & 0.691 & \cdots & 0.384 & 0.378 \\ 0.691 & 1 & \cdots & 0.383 & 0.377 \\ \vdots & \vdots & \ddots & & \\ 0.384 & 0.383 & \cdots & 1 & 0.210 \\ 0.378 & 0.377 & \cdots & 0.210 & 1 \end{pmatrix}_{100\times100}$

3. Simulation results for the selection of k

In this part, we compare the performances including the type I error rates and powers of two test statistics under the situations of $k = 2$ and $k = 3$, that is $\text{MAX}_2 = \max_{m_1+m_2=m} \xi_{m_1 m_2}$ and $\text{MAX}_3 = \max_{m_1+m_2+m_3=m} \xi_{m_1 m_2 m_3}$. 20 correlated phenotypes are simulated from the indirect and direct association models, respectively. Following the notations in the main text. For indirect association model, we assume that there are $L = 5$ latent factors and a genetic marker. Then Model 1 becomes $Y_i = U_{\lceil i/4 \rceil}\gamma_i + \varepsilon_i$, $U_{\lceil i/4 \rceil} = G\beta_{\lceil i/4 \rceil} + e_{\lceil i/4 \rceil}$, $i = 1, 2, \cdots, m$, where $\lceil i/4 \rceil$ denote the smallest integer that is greater than $i/4$, and $r_{i_1} = r_{i_2}$ if $\lceil i_1/4 \rceil = \lceil i_2/4 \rceil$, $i_1, i_2 \in \{1, 2, \cdots, m\}$. Without loss of generality, we assume that every four phenotypes subject to one common latent factor and the coefficients of latent factor are uniformly equal to 0.5 or 2. Hence, the derived correlation matrix of the simulated phenotypes under the null hypothesis ($\beta_1 = \beta_2 \cdots = \beta_5 = 0$) is 5-block diagonal with non-zero entry uniformly being equal to 0.2 or 0.8 which correspond to the first two patterns of correlation structures (uniform low correlation and uniform strong correlation)in the main text. Denote these two correlation structures as ST1 and ST2, respectively.

In the direct association model, we consider a scheme with one latent factor and a genetic marker. Model 2 can be written as $Y_i = U\gamma_i + G\beta_i + \varepsilon_i$, $i = 1, 2, \cdots, m$. Let all γ_i be 0.5 or 2 so that we also can obtain two kinds of correlation matrix (denoted by ST3 and ST4):

ST3. $\Delta = \left(\delta_{st}\right)_{20\times 20}$, $\delta_{ss} = 1, \delta_{st} = 0.2$, $s \neq t$, $s, t = 1, 2 \cdots, 20$;

ST4. $\Delta = \left(\delta_{st}\right)_{20\times 20}$, $\delta_{ss} = 1, \delta_{st} = 0.8$, $s \neq t$, $s, t = 1, 2 \cdots, 20$.

We consider six proportions: $\lambda = 0\%$, 20%, 40%, 60%, 80%, and 100% of the phenotypes are associated with the genotype G and MAF $\in \{0.05, 0.15, 0.30, 0.50\}$. $n = 1,500$ and $1,000$ are specified for MAF $= \{0.05, 0.15\}$ and MAF $\in \{0.30, 0.50\}$, respectively. We conduct 10,000 bootstraps to calculate the empirical distribution of MAX_2 and MAX_3. The proportions of the variance of the associated phenotypes explained by the genetic variant are 0.1% when the empirical powers are calculated. The empirical type I error rates and powers are calculated based on 1,000 replicates.

Table S3 and Table S4 show the empirical type I error rates and power of MAX_2 and MAX_3 under the nominal significance level of 0.05 for indirect and direct association models, respectively. From both tables, we find that both MAX_2 and MAX_3 have correct type I error rates. For example, When the correlation structure is ST2 and MAF = 0.30, and the trait are simulated from Model 2, the empirical type I error rates of MAX_2 and MAX_3 are 0.049 and 0.048, respectively. MAX_2 is more powerful than MAX_3 in most sce-

narios, especially when the number of associated phenotypes is large. For example, when MAF $= 0.15, n = 1,000$ and the trait are simulated from Model 2 with the correlation structure of ST3, the powers of MAX_2 for five levels associations (20%, 40%, 60%, 80%, and 100%) are 0.192, 0.361, 0.446, 0.460, and 0.484, which are respectively greater than 0.209, 0.355, 0.411, 0.430, and 0.396 of MAX_3.

Table S3. The empirical type I errors and power of MAX_2 and MAX_3 for 20 correlated phenotypes sampled from Model 1 with the correlation structures ST1 and ST2. The nominal significance level of 0.05 and 1,000 replicates are conducted.

		MAF = 0.05		MAF = 0.15		MAF = 0.30		MAF = 0.50	
	λ	MAX_2	MAX_3	MAX_2	MAX_3	MAX_2	MAX_3	MAX_2	MAX_3
ST1	0%	0.053	0.063	0.072	0.063	0.053	0.052	0.042	0.041
	20%	0.168	0.158	0.194	0.176	0.125	0.124	0.120	0.117
	40%	0.401	0.376	0.382	0.364	0.252	0.243	0.248	0.204
	60%	0.569	0.546	0.557	0.555	0.370	0.338	0.365	0.366
	80%	0.746	0.725	0.725	0.709	0.526	0.492	0.519	0.493
	100%	0.836	0.816	0.840	0.826	0.640	0.623	0.613	0.598
ST2	0%	0.044	0.047	0.053	0.052	0.049	0.048	0.056	0.050
	20%	0.094	0.088	0.108	0.094	0.087	0.089	0.076	0.080
	40%	0.191	0.165	0.170	0.168	0.114	0.112	0.129	0.115
	60%	0.257	0.250	0.263	0.257	0.180	0.179	0.180	0.167
	80%	0.335	0.325	0.333	0.317	0.220	0.198	0.241	0.214
	100%	0.437	0.429	0.446	0.430	0.307	0.278	0.310	0.283

Table S4. The empirical type I errors and power of MAX_2 and MAX_3 for 20 correlated phenotypes sampled from Model 2 with the correlation structures ST3 and ST4. The nominal significance level of 0.05 and 1,000 replicates are conducted.

		MAF = 0.05		MAF = 0.15		MAF = 0.30		MAF = 0.50	
	λ	MAX_2	MAX_3	MAX_2	MAX_3	MAX_2	MAX_3	MAX_2	MAX_3
ST3	0%	0.059	0.050	0.045	0.054	0.049	0.041	0.049	0.050
	20%	0.202	0.218	0.192	0.209	0.155	0.150	0.140	0.135
	40%	0.383	0.375	0.361	0.355	0.239	0.243	0.232	0.228
	60%	0.425	0.409	0.446	0.411	0.283	0.280	0.286	0.257
	80%	0.443	0.395	0.460	0.430	0.334	0.296	0.310	0.274
	100%	0.447	0.345	0.484	0.396	0.304	0.240	0.305	0.258
ST4	0%	0.052	0.044	0.043	0.050	0.051	0.043	0.054	0.059
	20%	0.817	0.833	0.791	0.810	0.576	0.610	0.585	0.620
	40%	0.962	0.967	0.967	0.969	0.816	0.826	0.836	0.836
	60%	0.974	0.975	0.978	0.977	0.838	0.846	0.826	0.844
	80%	0.849	0.835	0.846	0.845	0.627	0.633	0.627	0.637
	100%	0.161	0.114	0.128	0.115	0.090	0.084	0.102	0.092

4. Simulation results when the associated phenotypes are selected with equal probability from indirect association model

Figure S1. The empirical power of five tests for 20 correlated phenotypes sampled from the indirect association Model (Model 1). The associated phenotypes are randomly selected with equal probability. 1,000 replicates are conducted under the nominal significant level of 0.05. Scenario S1-S4 correspond to four patterns of correlation structures for m=20. The proportions of the variance explained by the genetic variance for S1-S4 are 0.1%, 0.2%, 0.1%, and 0.2%, respectively. λ is the proportion of phenotypes that are subject to the genetic variant.

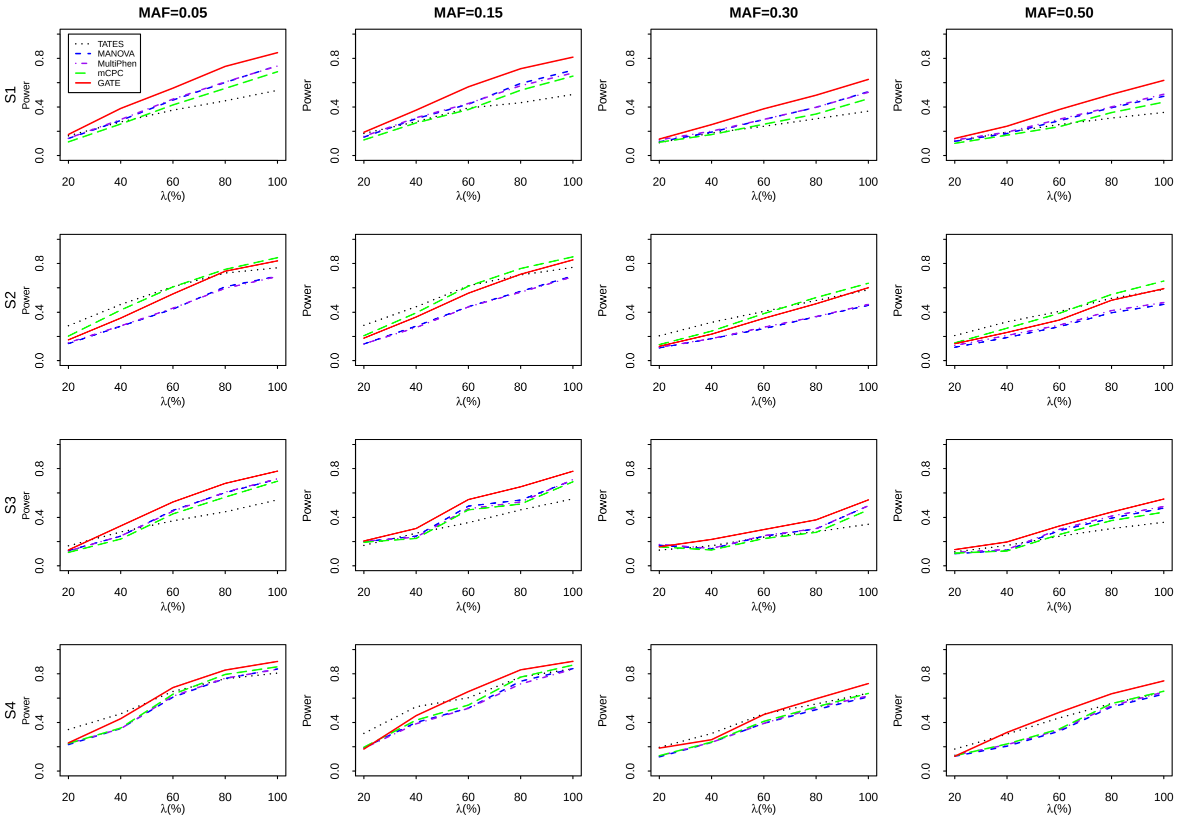

Figure S2. The empirical power of four tests for 100 correlated phenotypes sampled from the indirect association Model. The associated phenotypes are randomly selected with equal probability. 1,000 replicates are conducted under the nominal significant level of 0.05. Scenario S5-S8 correspond to four patterns of correlation structures for m=100. The proportions of the variance explained by the genetic variance for S5-S8 are 0.1%, 0.1%, 0.05%, and 0.1%, respectively. λ is the proportion of phenotypes that are subject to the genetic variant.

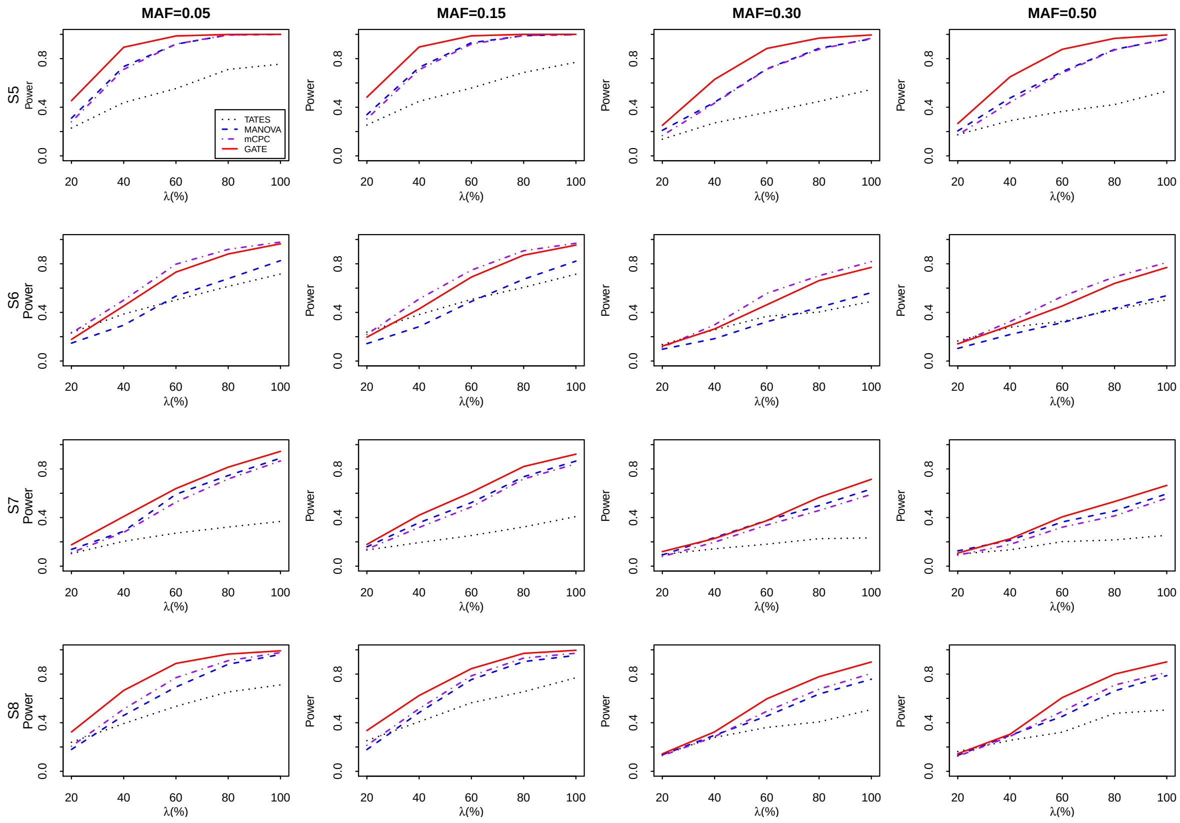

5. Simulation results when the associated phenotypes are selected with equal probability from direct association model

Figure S3. The empirical power of five tests for 20 correlated phenotypes sampled from the direct association Model. The associated phenotypes are randomly selected with equal probability. 1,000 replicates are conducted under the nominal significant level of 0.05. Scenario S9-S12 correspond to four patterns of correlation structures for m=20. The proportions of the variance explained by the genetic variance for S9-S12 are 0.2%, 0.1%, 0.2%, and 0.2%, respectively. λ is the proportion of phenotypes that are subject to the genetic variant.

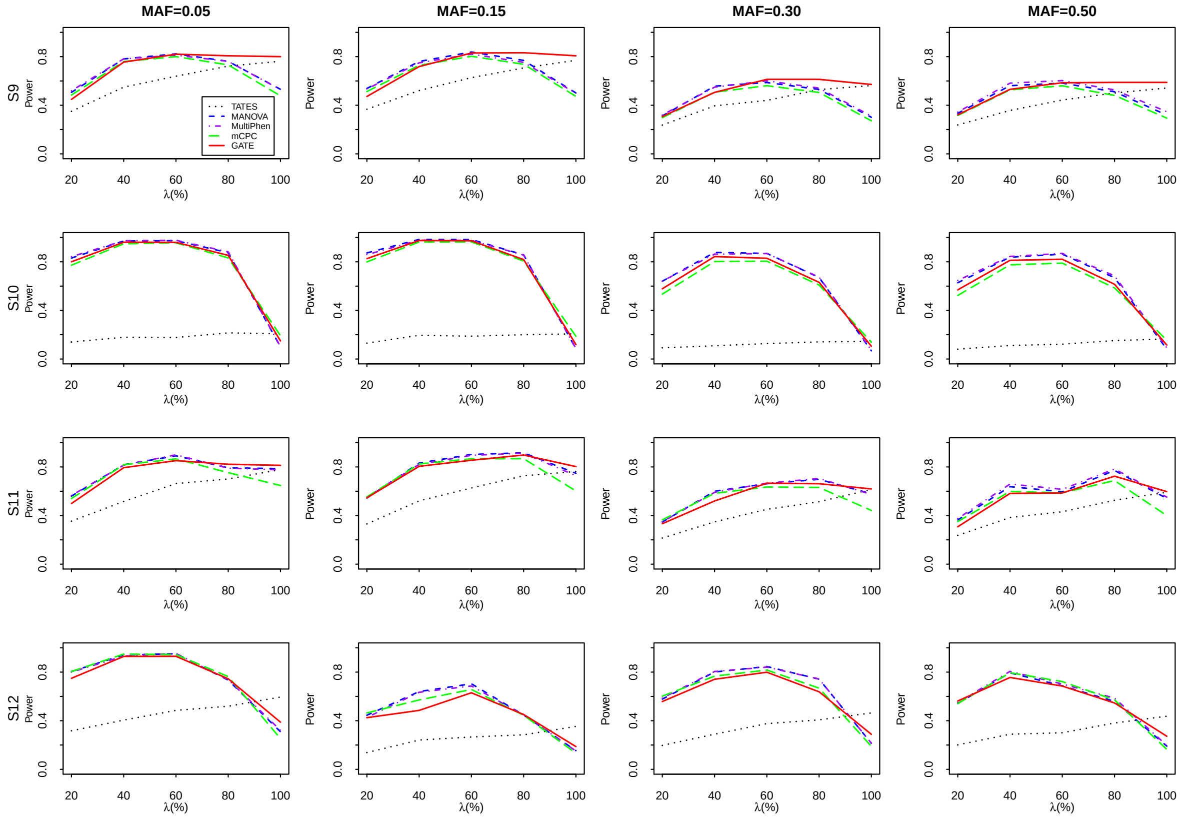

Figure S4. The empirical power of four tests for 100 correlated phenotypes sampled from the direct association Model. The associated phenotypes are randomly selected with equal probability. 1,000 replicates are conducted under the nominal significant level of 0.05. Scenario S13-S16 correspond to four patterns of correlation structures for m=100. The proportions of the variance explained by the genetic variance for S13-S16 are all equal to 0.1%. λ is the proportion of phenotypes that are subject to the genetic variant.

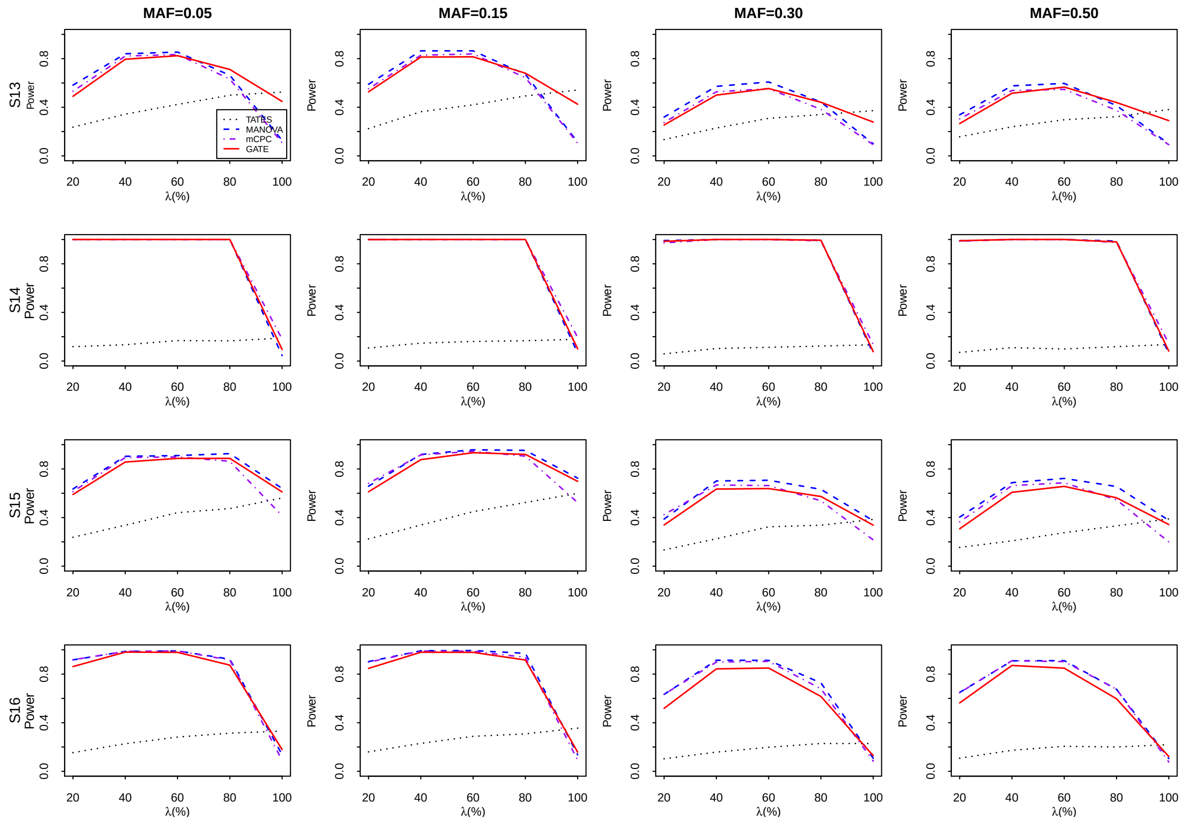

6. Simulation results for $m = 10$.

6.1. Simulation settings for Indirect and direct association models when $m = 10$.

When $m = 10$, we also consider four patterns of correlation structures: (1) uniform low correlation; (2) uniform strong correlation; (3) a gradient of moderate to low correlations; (4) a gradient of strong to moderate correlations. The detailed settings of γ_i,

$i = 1, 2\cdots, m$ corresponding to these four correlation structures in the Indirect trait model for $m = 10$ are presented as follows :

S17. $\tilde{\gamma} = (0.5, 0.5, 0.5)^\tau$; $\Delta_1 = \Delta_2 = \begin{pmatrix} 1 & 0.2 & 0.2 & 0.2 \\ 0.2 & 1 & 0.2 & 0.2 \\ 0.2 & 0.2 & 1 & 0.2 \\ 0.2 & 0.2 & 0.2 & 1 \end{pmatrix}$ and $\Delta_3 = \begin{pmatrix} 1 & 0.2 \\ 0.2 & 1 \end{pmatrix}$;

S18. $\tilde{\gamma} = (2.0, 2.0, 2.0)^\tau$; $\Delta_1 = \Delta_2 = \begin{pmatrix} 1 & 0.8 & 0.8 & 0.8 \\ 0.8 & 1 & 0.8 & 0.8 \\ 0.8 & 0.8 & 1 & 0.8 \\ 0.8 & 0.8 & 0.8 & 1 \end{pmatrix}$ and $\Delta_3 = \begin{pmatrix} 1 & 0.8 \\ 0.8 & 1 \end{pmatrix}$;

S19. $\tilde{\gamma} = (1.0, 0.8, 0.6)^\tau$; $\Delta_1 = (\delta_{st}^{(1)})_{4\times4}$, $\delta_{ss}^{(1)} = 1$, $\delta_{st}^{(1)} = 0.500$ when $s \neq t$; $\Delta_2 = (\delta_{st}^{(2)})_{4\times4}$, $\delta_{ss}^{(2)} = 1, \delta_{st}^{(2)} = 0.390$, when $s \neq t$; $\Delta_3 = (\delta_{st}^{(3)})_{2\times2}$, $\delta_{ss}^{(3)} = 1, \delta_{st}^{(3)} = 0.265$ when $s \neq t$;

S20. $\tilde{\gamma} = (1.5, 1.3, 1.1)^\tau$; $\Delta_1 = (\delta_{st}^{(1)})_{4\times4}$, $\delta_{ss}^{(1)} = 1, \delta_{st}^{(1)} = 0.692$ when $s \neq t$; $\Delta_2 = (\delta_{st}^{(2)})_{4\times4}$, $\delta_{ss}^{(2)} = 1, \delta_{st}^{(2)} = 0.628$ when $s \neq t$; $\Delta_3 = (\delta_{st}^{(3)})_{2\times2}$, $\delta_{ss}^{(3)} = 1, \delta_{st}^{(3)} = 0.548$ when $s \neq t$.

In addition, the detailed settings of γ_i, $i = 1, 2\cdots, m$, corresponding to these four correlation structures in the direct trait model for $m = 10$ are

S21. $\gamma_i = 0.50$, $i = 1, 2, \cdots, 10$; $\Delta = \Big(\delta_{st}\Big)_{10\times10}, \delta_{ss} = 1$, $\delta_{st} = 0.2$ when $s \neq t$;

S22. $\gamma_i = 2.00$, $i = 1, 2, \cdots, 10$; $\Delta = \Big(\delta_{st}\Big)_{10\times10}, \delta_{ss} = 1$, $\delta_{st} = 0.8$ when $s \neq t$;

S23. $\gamma_i = 1.00 - 0.05(i-1), i = 1, 2, \cdots, 10$; $\Delta = \begin{pmatrix} 1 & 0.480 & \cdots & 0.341 \\ 0.480 & 1 & \cdots & 0.331 \\ \vdots & \vdots & \ddots & \\ 0.341 & 0.331 & \cdots & 1 \end{pmatrix}_{10\times10}$;

S24. $\gamma_i = 1.50 - 0.05(i-1), i = 1, 2, \cdots, 10$; $\Delta = \begin{pmatrix} 1 & 0.680 & \cdots & 0.602 \\ 0.680 & 1 & \cdots & 0.596 \\ \vdots & \vdots & \ddots & \\ 0.602 & 0.596 & \cdots & 1 \end{pmatrix}_{10\times10}$.

6.2. Simulation results for $m = 10$.

Table S5. The empirical type I errors of TATES, MANOVA, MultiPhen, mCPC, and GATE when the correlated phenotypes are sampled from indirect association model. The number of correlated phenotypes is 10. Scenario S17-S20 correspond to four correlation structures for the Indirect association model and Scenario S21-S24 are for the direct association model. For each scenario, four MAFs including 0.05, 0.15, 0.30, and 0.50 are considered. The nominal significance level is 0.05 and 1000 replicates are conducted.

	Scenario	MAF	TATES	MANOVA	MultiPhen	mCPC	GATE
Indirect Model	S17	0.05	0.057	0.046	0.053	0.044	0.051
		0.15	0.049	0.053	0.056	0.052	0.047
		0.30	0.044	0.053	0.061	0.053	0.051
		0.50	0.053	0.057	0.059	0.058	0.053
	S18	0.05	0.056	0.048	0.050	0.045	0.054
		0.15	0.052	0.042	0.047	0.045	0.048
		0.30	0.060	0.049	0.045	0.051	0.054
		0.50	0.066	0.048	0.051	0.050	0.058
	S19	0.05	0.058	0.059	0.057	0.053	0.055
		0.15	0.049	0.048	0.049	0.051	0.054
		0.30	0.048	0.056	0.061	0.051	0.050
		0.50	0.060	0.054	0.056	0.056	0.053
	S20	0.05	0.055	0.058	0.061	0.055	0.046
		0.15	0.060	0.058	0.057	0.059	0.061
		0.30	0.054	0.049	0.054	0.049	0.051
		0.50	0.051	0.047	0.048	0.052	0.046
Direct Model	S21	0.05	0.046	0.043	0.044	0.044	0.044
		0.15	0.052	0.042	0.046	0.046	0.044
		0.30	0.052	0.050	0.050	0.051	0.055
		0.50	0.052	0.045	0.047	0.042	0.047
	S22	0.05	0.044	0.046	0.048	0.048	0.052
		0.15	0.049	0.065	0.061	0.057	0.056
		0.30	0.037	0.046	0.049	0.048	0.052
		0.50	0.049	0.053	0.055	0.054	0.051
	S23	0.05	0.052	0.054	0.057	0.055	0.057
		0.15	0.054	0.049	0.049	0.049	0.052
		0.30	0.060	0.053	0.058	0.054	0.054
		0.50	0.051	0.053	0.055	0.053	0.053
	S24	0.05	0.043	0.048	0.049	0.050	0.044
		0.15	0.045	0.052	0.049	0.054	0.053
		0.30	0.053	0.049	0.050	0.054	0.053
		0.50	0.039	0.060	0.063	0.057	0.057

Figure S5. The empirical power of five tests for 10 correlated phenotypes sampled from the Indirect association model with correlation structure S17-S20. 1,000 replicates are conducted under the nominal significant level of 0.05.

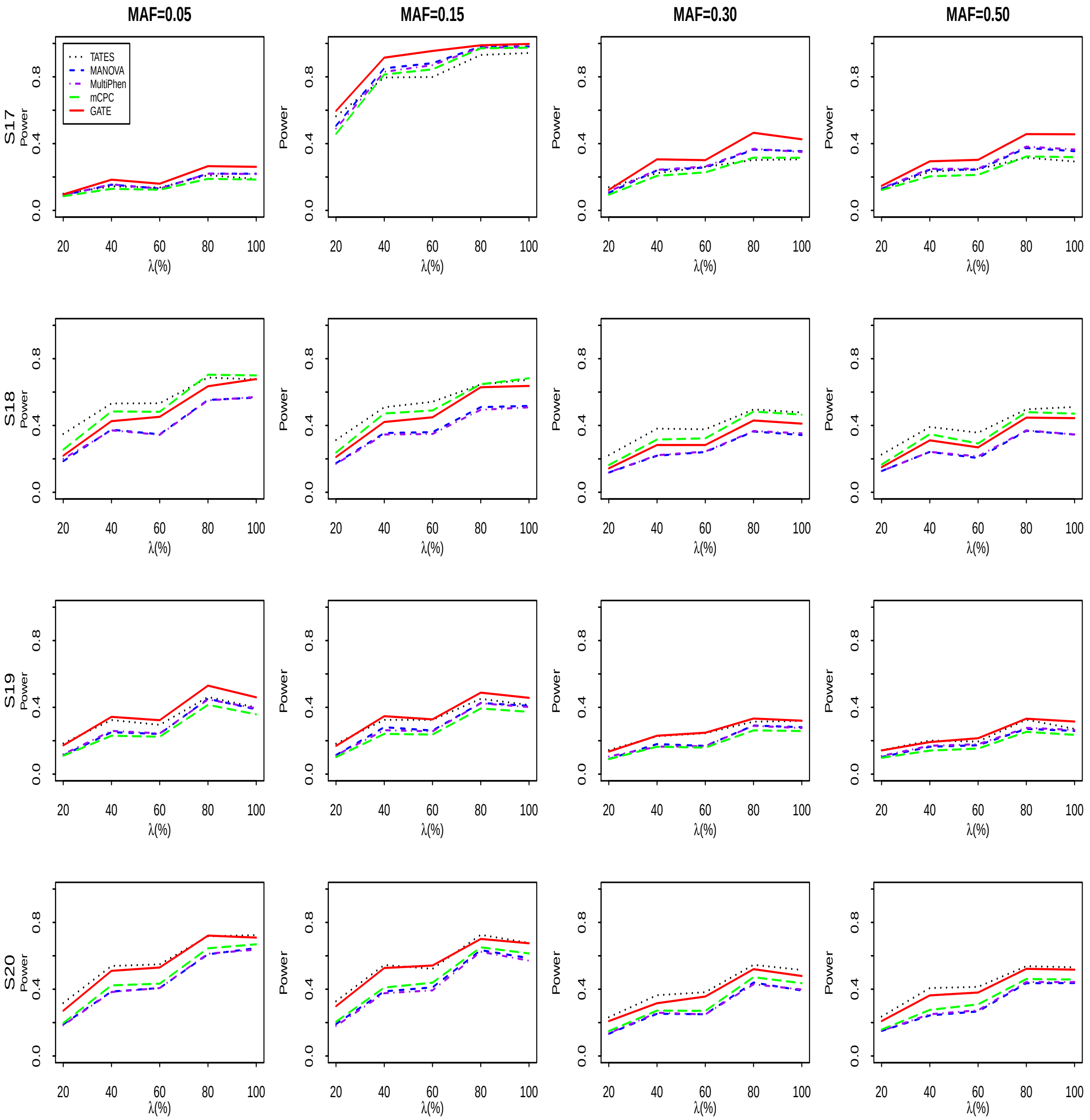

Figure S6. The empirical power of five tests for 10 correlated phenotypes sampled from the direct association model with correlation structure S21-S24. 1,000 replicates are conducted under the nominal significant level of 0.05.

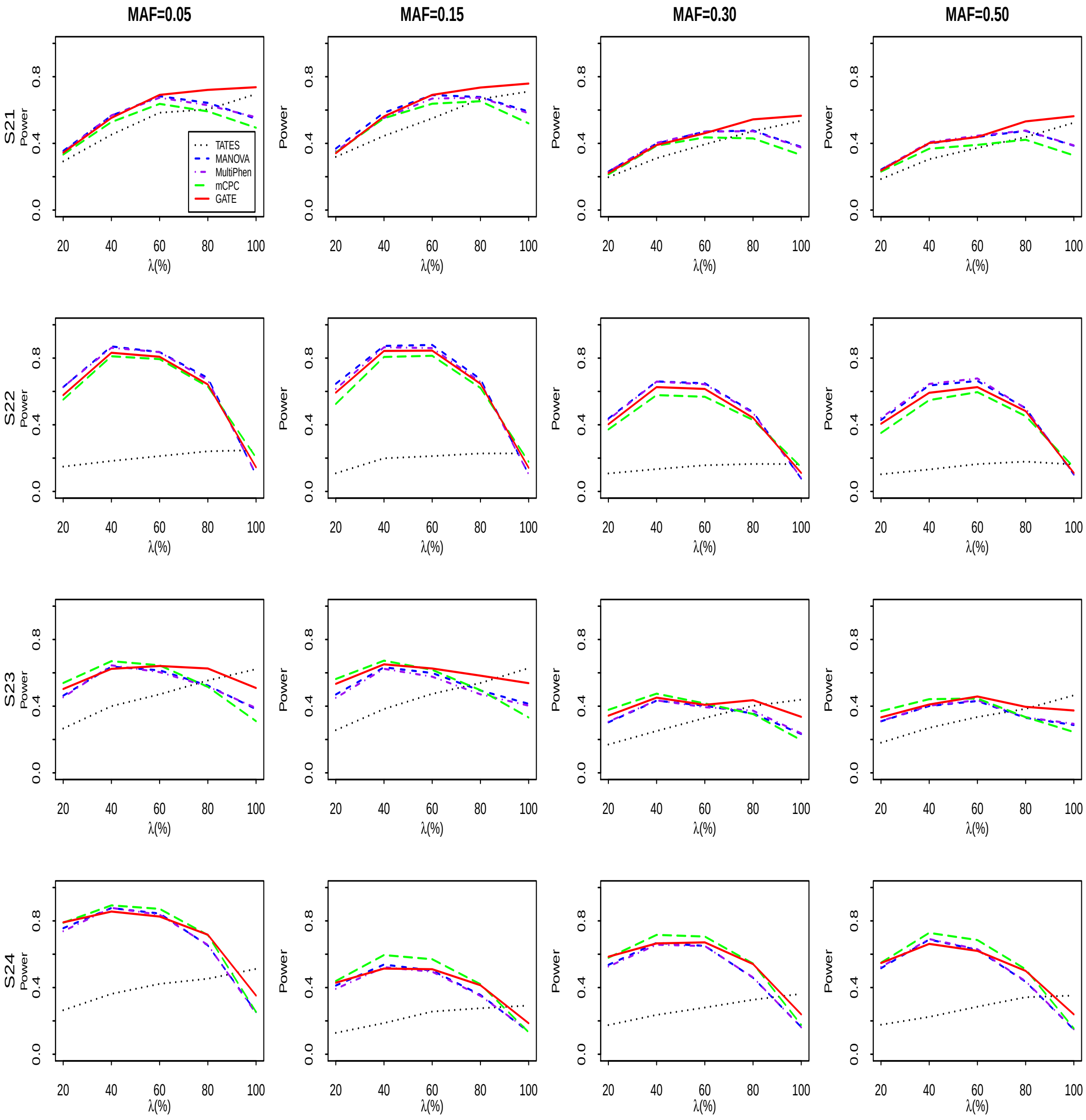

7. Simulation results for $m = 50$.

7.1. Simulation settings for Indirect and direct association models when $m = 50$.

Similarly, we consider four correlation structures for $m = 50$. Assume that $\gamma_i = \gamma_j$ if $\lceil i/4 \rceil = \lceil j/4 \rceil$, so there are $L = 13$ different values for γ_i, $i = 1, 2 \cdots, m$, which we denote as $\tilde{\gamma} = (\tilde{\gamma}_1, \tilde{\gamma}_2, \cdots, \tilde{\gamma}_{13})^\tau$. The detailed settings of $\tilde{\gamma}_i$, $i = 1, 2 \cdots, 13$, corresponding to these four correlation structures in the Indirect trait model for $m = 50$ are presented as follows:

S25. $\tilde{\gamma}_i = 0.50,\ i = 1, 2, \cdots, 13;\ \ \Delta_1 = \cdots = \Delta_{12} = \begin{pmatrix} 1 & 0.2 & 0.2 & 0.2 \\ 0.2 & 1 & 0.2 & 0.2 \\ 0.2 & 0.2 & 1 & 0.2 \\ 0.2 & 0.2 & 0.2 & 1 \end{pmatrix}$ and $\Delta_{13} = \begin{pmatrix} 1 & 0.2 \\ 0.2 & 1 \end{pmatrix}$;

S26. $\tilde{\gamma}_i = 2.0,\ i = 1, 2, \cdots, 13;\ \ \Delta_1 = \cdots = \Delta_{12} = \begin{pmatrix} 1 & 0.8 & 0.8 & 0.8 \\ 0.8 & 1 & 0.8 & 0.8 \\ 0.8 & 0.8 & 1 & 0.8 \\ 0.8 & 0.8 & 0.8 & 1 \end{pmatrix}$ and $\Delta_{13} = \begin{pmatrix} 1 & 0.8 \\ 0.8 & 1 \end{pmatrix}$;

S27. $\tilde{\gamma}_i = 1 - 0.04(i-1), i = 1, 2, \cdots, 13;\ \ \Delta_1 = (\delta_{st}^{(1)})_{4\times4},\ \delta_{ss}^{(1)} = 1, \delta_{st}^{(1)} = 0.500,$ when $s \neq t; \Delta_2 = (\delta_{st}^{(2)})_{4\times4},\ \delta_{ss}^{(2)} = 1, \delta_{st}^{(2)} = 0.480,$ when $s \neq t; \cdots \Delta_{12} = (\delta_{st}^{(12)})_{4\times4},\ \delta_{ss}^{(12)} = 1, \delta_{st}^{(12)} = 0.238,$ when $s \neq t; \Delta_{13} = (\delta_{st}^{(13)})_{2\times2},\ \delta_{ss}^{(13)} = 1, \delta_{st}^{(13)} = 0.212,$ when $s \neq t$;

S28. $\tilde{\gamma}_i = 1.5 - 0.04(i-1), i = 1, 2, \cdots, 13;\ \ \Delta_1 = (\delta_{st}^{(1)})_{4\times4},\ \delta_{ss}^{(1)} = 1, \delta_{st}^{(1)} = 0.692,$ when $s \neq t; \Delta_2 = (\delta_{st}^{(2)})_{4\times4},\ \delta_{ss}^{(2)} = 1, \delta_{st}^{(2)} = 0.686,$ when $s \neq t; \cdots \Delta_{12} = (\delta_{st}^{(12)})_{4\times4},\ \delta_{ss}^{(12)} = 1, \delta_{st}^{(12)} = 0.529,$ when $s \neq t; \Delta_{13} = (\delta_{st}^{(13)})_{2\times2},\ \delta_{ss}^{(13)} = 1, \delta_{st}^{(13)} = 0.519,$ when $s \neq t$;

Besides, the detailed settings of γ_i, $i = 1, 2 \cdots, m$ corresponding to these four correlation structures in the direct trait model for $m = 50$ are

S29. $\gamma_i = 0.50,\ i = 1, 2, \cdots, 50;\ \ \Delta = \Big(\delta_{st}\Big)_{50\times50}, \delta_{ss} = 1,\ \delta_{st} = 0.2$ when $s \neq t$;

S30. $\gamma_i = 2.00,\ i = 1, 2, \cdots, 50;\ \ \Delta = \Big(\delta_{st}\Big)_{50\times50}, \delta_{ss} = 1,\ \delta_{st} = 0.8$ when $s \neq t$;

S31. $\gamma_i = 1.00 - 0.05(i-1), i = 1, 2, \cdots, 50;\ \ \Delta = \begin{pmatrix} 1 & 0.480 & \cdots & 0.341 \\ 0.480 & 1 & \cdots & 0.331 \\ \vdots & \vdots & \ddots & \\ 0.341 & 0.331 & \cdots & 1 \end{pmatrix}_{50\times50}$;

S32. $\gamma_i = 1.50 - 0.05(i-1), i = 1, 2, \cdots, 50;\ \ \Delta = \begin{pmatrix} 1 & 0.680 & \cdots & 0.602 \\ 0.680 & 1 & \cdots & 0.596 \\ \vdots & \vdots & \ddots & \\ 0.602 & 0.596 & \cdots & 1 \end{pmatrix}_{50\times 50}.$

7.2. Simulation results for $m = 50$.

Table S6. The empirical type I errors of TATES, MANOVA, MultiPhen, mCPC, and GATE when the correlated phenotypes are sampled from indirect association model. The number of correlated phenotypes is 50. Scenario S25-S28 correspond to four correlation structures for the Indirect association model and Scenario S29-S32 are for the direct association model. For each scenario, four MAFs including 0.05, 0.15, 0.30, and 0.50 are considered. The nominal significance level is 0.05 and 1000 replicates are conducted.

	Scenario	MAF	TATES	MANOVA	MultiPhen	mCPC	GATE
Indirect Model	S25	0.05	0.044	0.054	0.070	0.051	0.041
		0.15	0.049	0.051	0.058	0.045	0.050
		0.30	0.051	0.051	0.072	0.049	0.051
		0.50	0.044	0.049	0.065	0.049	0.037
	S26	0.05	0.044	0.055	0.068	0.052	0.051
		0.15	0.052	0.047	0.065	0.045	0.047
		0.30	0.049	0.051	0.071	0.048	0.054
		0.50	0.056	0.049	0.063	0.056	0.051
	S27	0.05	0.048	0.057	0.067	0.055	0.053
		0.15	0.056	0.048	0.059	0.054	0.051
		0.30	0.054	0.052	0.071	0.051	0.050
		0.50	0.059	0.054	0.066	0.056	0.053
	S28	0.05	0.05	0.044	0.063	0.052	0.048
		0.15	0.15	0.049	0.075	0.056	0.061
		0.30	0.30	0.037	0.062	0.052	0.049
		0.50	0.50	0.049	0.073	0.051	0.055
Direct Model	S29	0.05	0.039	0.039	0.052	0.036	0.047
		0.15	0.048	0.060	0.071	0.054	0.055
		0.30	0.053	0.050	0.074	0.053	0.060
		0.50	0.053	0.048	0.067	0.043	0.051
	S30	0.05	0.046	0.050	0.063	0.041	0.046
		0.15	0.038	0.050	0.062	0.044	0.053
		0.30	0.044	0.051	0.074	0.047	0.044
		0.50	0.050	0.043	0.059	0.040	0.051
	S31	0.05	0.062	0.051	0.067	0.052	0.048
		0.15	0.056	0.059	0.073	0.060	0.056
		0.30	0.045	0.044	0.060	0.050	0.043
		0.50	0.055	0.049	0.067	0.044	0.053
	S32	0.05	0.043	0.046	0.064	0.055	0.058
		0.15	0.046	0.045	0.068	0.047	0.042
		0.30	0.033	0.044	0.071	0.040	0.043
		0.50	0.054	0.055	0.074	0.057	0.057

Figure S7. The empirical power of five tests for 50 correlated phenotypes sampled from the Indirect association model with correlation structure S25-S28. 1,000 replicates are conducted under the nominal significant level of 0.05.

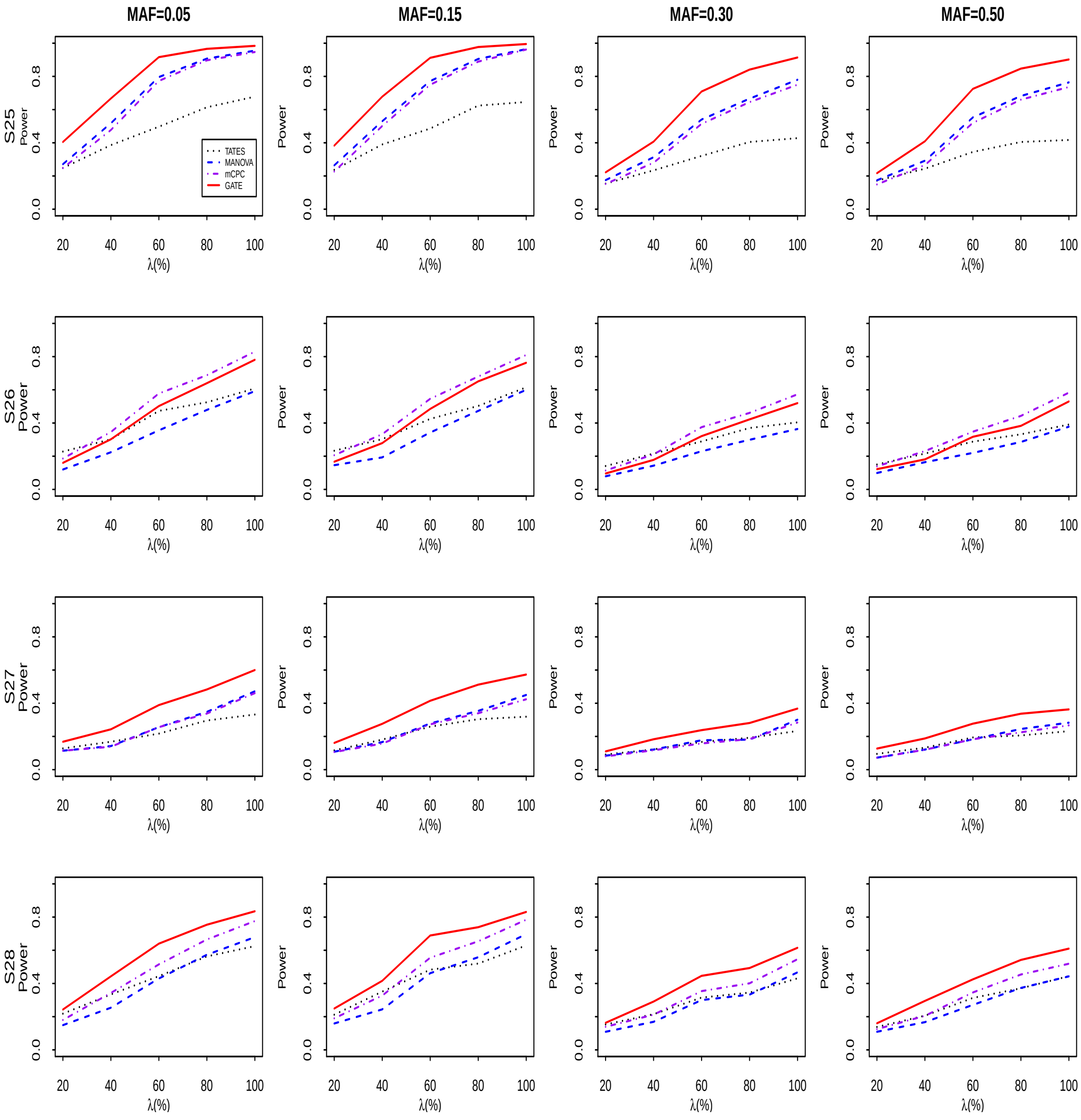

Figure S8. The empirical power of five tests for 50 correlated phenotypes sampled from the Indirect association model with correlation structure S29-S32. 1,000 replicates are conducted under the nominal significant level of 0.05.

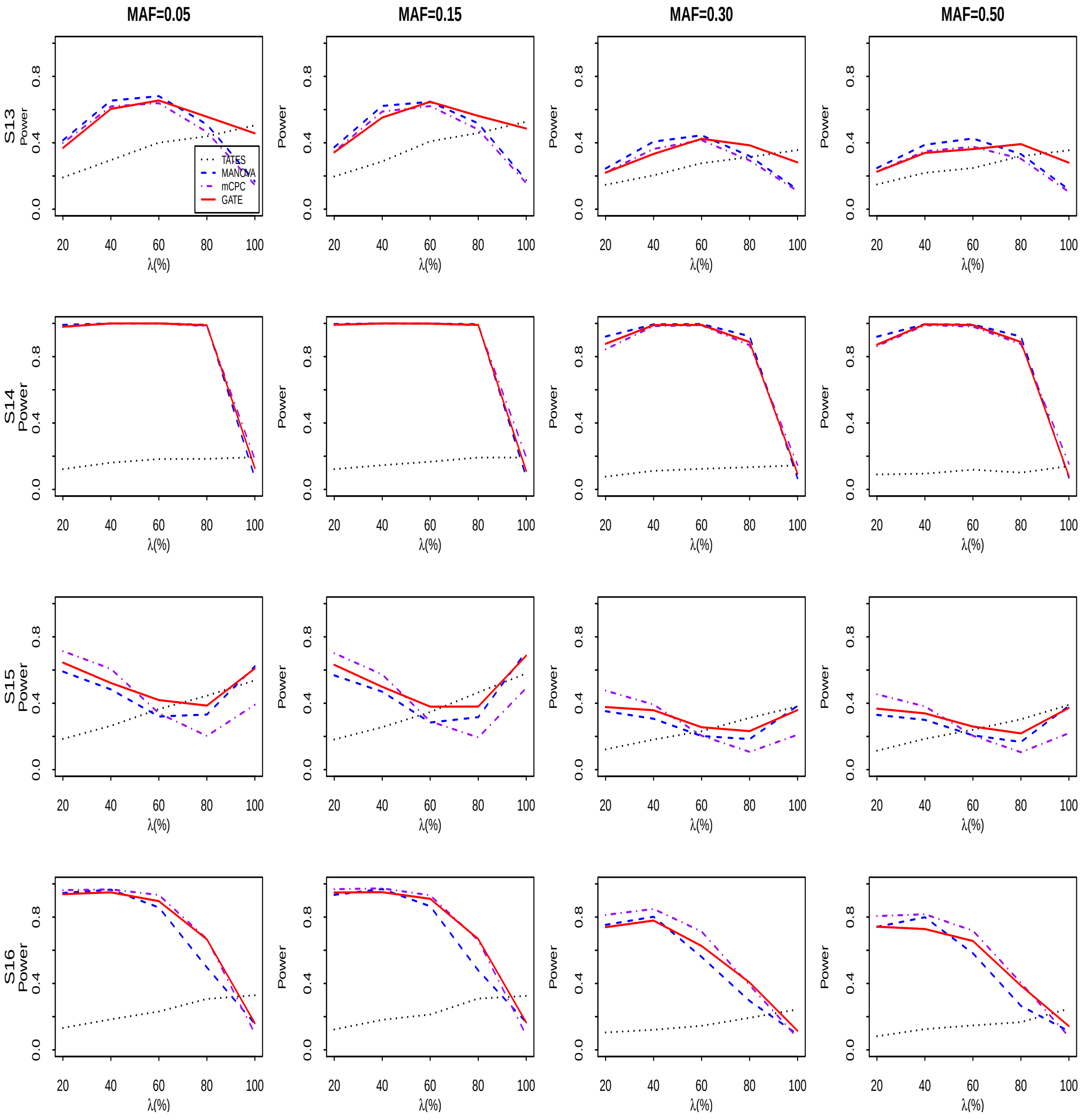

8. The estimation of density function of the GATE considering $k \in \{1, 2\}$.

Table S7. The quantiles of the distribution of $-2\log(\text{GATE})$ for $k \in \{1, 2\}$ when the dimension m ranges from 10 to 200. The empirical quantiles of 0.95, 0.99, 0.999, 0.9999, and 0.99999 based on 1,000,000 replicates are in the 2-6th column. The corresponding estimated quantiles are obtained in the 10-14th column using the generalized Gamma distribution with scale parameter a, shape parameter b and κ.

	Empirical Distribution					Generalized Gamma distribution							
	Quantiles					Parameter			Quantiles				
m	95%	99%	99.9%	99.99%	99.999%	a	b	κ	95%	99%	99.9%	99.99%	99.999%
10	7.416	10.812	15.571	20.267	26.054	1.147	0.857	2.131	7.421	10.884	15.872	20.937	26.086
11	7.521	10.931	15.614	20.451	25.561	1.112	0.850	2.206	7.514	11.001	16.027	21.134	26.329
12	7.562	10.956	15.742	20.502	24.590	1.116	0.854	2.242	7.567	11.034	16.015	21.064	26.192
13	7.654	11.047	15.811	20.655	24.532	1.091	0.849	2.302	7.646	11.132	16.142	21.221	26.383
14	7.708	11.107	15.943	20.812	25.907	1.079	0.848	2.349	7.707	11.195	16.201	21.274	26.426
15	7.744	11.160	15.989	20.946	25.907	1.060	0.846	2.401	7.751	11.237	16.239	21.307	26.453
16	7.812	11.227	15.985	20.673	25.831	1.045	0.843	2.445	7.803	11.298	16.309	21.384	26.540
17	7.879	11.297	16.117	20.784	24.973	1.019	0.838	2.505	7.867	11.378	16.415	21.520	26.709
18	7.894	11.341	16.168	20.980	25.749	1.043	0.845	2.500	7.906	11.405	16.411	21.472	26.606
19	7.962	11.409	16.142	20.436	24.687	1.018	0.840	2.557	7.956	11.465	16.488	21.569	26.726
20	8.008	11.457	16.250	21.342	26.010	1.000	0.836	2.606	8.007	11.526	16.564	21.662	26.837
21	8.039	11.481	16.228	21.006	26.210	1.015	0.841	2.602	8.035	11.547	16.565	21.635	26.777
22	8.071	11.511	16.433	21.318	26.145	0.996	0.837	2.650	8.072	11.591	16.619	21.700	26.854
23	8.131	11.580	16.427	21.067	25.001	0.982	0.834	2.686	8.128	11.667	16.726	21.843	27.036
24	8.154	11.617	16.405	21.041	24.983	0.978	0.834	2.715	8.155	11.687	16.732	21.831	27.002
25	8.212	11.713	16.522	21.124	25.892	0.942	0.825	2.779	8.202	11.763	16.858	22.017	27.259
26	8.215	11.690	16.445	21.471	25.068	0.968	0.832	2.760	8.219	11.760	16.815	21.920	27.098
27	8.243	11.711	16.569	20.904	24.914	0.961	0.832	2.788	8.241	11.778	16.822	21.916	27.081
28	8.287	11.755	16.491	21.223	25.491	0.956	0.831	2.813	8.279	11.824	16.880	21.984	27.159
29	8.298	11.770	16.607	21.413	26.689	0.938	0.828	2.854	8.297	11.843	16.902	22.010	27.191
30	8.330	11.829	16.595	21.008	24.727	0.923	0.824	2.889	8.324	11.880	16.956	22.085	27.290
31	8.374	11.865	16.729	21.739	25.590	0.917	0.823	2.916	8.370	11.937	17.027	22.170	27.389
32	8.368	11.863	16.680	21.480	26.493	0.923	0.826	2.920	8.374	11.927	16.991	22.102	27.285
33	8.405	11.884	16.697	21.167	24.829	0.949	0.832	2.893	8.403	11.951	16.997	22.082	27.231
34	8.441	11.909	16.865	21.810	26.451	0.925	0.827	2.944	8.433	11.993	17.061	22.173	27.354
35	8.455	11.926	16.705	21.606	25.895	0.930	0.828	2.949	8.447	12.001	17.057	22.152	27.312

Table S7. *continued*

	Empirical Distribution					Generalized Gamma distribution							
	Quantiles					Parameter			Quantiles				
m	95%	99%	99.9%	99.99%	99.999%	a	b	κ	95%	99%	99.9%	99.99%	99.999%
36	8.478	11.969	16.853	21.481	26.003	0.920	0.826	2.974	8.473	12.037	17.108	22.221	27.401
37	8.488	11.988	16.819	21.555	26.185	0.903	0.823	3.018	8.484	12.043	17.107	22.214	27.389
38	8.522	11.999	16.781	21.745	26.549	0.880	0.818	3.066	8.511	12.085	17.177	22.318	27.532
39	8.549	12.086	16.979	21.878	26.238	0.873	0.815	3.088	8.554	12.148	17.270	22.443	27.693
40	8.570	12.072	16.910	21.484	26.735	0.897	0.822	3.059	8.560	12.137	17.224	22.352	27.549
41	8.590	12.118	16.897	21.892	26.433	0.904	0.824	3.061	8.580	12.153	17.231	22.346	27.527
42	8.601	12.071	16.897	21.615	26.805	0.898	0.823	3.081	8.592	12.165	17.241	22.353	27.531
43	8.650	12.139	16.983	21.758	26.237	0.882	0.818	3.116	8.633	12.229	17.343	22.500	27.727
44	8.645	12.159	16.968	21.877	25.837	0.891	0.821	3.111	8.638	12.223	17.316	22.447	27.643
45	8.660	12.165	17.068	22.085	26.901	0.871	0.817	3.154	8.655	12.250	17.361	22.514	27.738
46	8.666	12.195	17.068	21.819	26.278	0.875	0.819	3.159	8.670	12.257	17.353	22.488	27.689
47	8.688	12.219	17.151	21.985	26.140	0.874	0.818	3.170	8.695	12.290	17.397	22.543	27.755
48	8.708	12.222	17.173	21.904	26.750	0.879	0.820	3.171	8.698	12.284	17.374	22.498	27.686
49	8.726	12.279	17.172	21.759	27.008	0.832	0.809	3.264	8.727	12.337	17.474	22.659	27.919
50	8.739	12.288	17.198	22.422	26.100	0.861	0.816	3.218	8.737	12.336	17.449	22.599	27.816
51	8.759	12.276	17.293	21.897	25.761	0.855	0.814	3.238	8.754	12.358	17.477	22.635	27.860
52	8.789	12.365	17.310	22.333	26.710	0.852	0.813	3.253	8.786	12.401	17.535	22.709	27.951
53	8.785	12.317	17.182	22.229	26.949	0.861	0.817	3.246	8.781	12.381	17.489	22.629	27.834
54	8.791	12.299	17.169	22.154	27.137	0.883	0.822	3.218	8.793	12.384	17.469	22.580	27.749
55	8.810	12.357	17.283	22.022	26.307	0.856	0.816	3.270	8.808	12.411	17.520	22.662	27.868
56	8.825	12.416	17.362	21.903	27.159	0.849	0.814	3.292	8.838	12.452	17.579	22.741	27.968
57	8.843	12.412	17.264	21.886	26.539	0.862	0.817	3.274	8.837	12.442	17.550	22.689	27.888
58	8.865	12.393	17.332	21.944	27.295	0.853	0.815	3.301	8.855	12.464	17.579	22.726	27.935
59	8.874	12.390	17.168	22.095	26.747	0.856	0.817	3.304	8.859	12.460	17.563	22.693	27.884
60	8.867	12.411	17.367	22.106	26.357	0.863	0.819	3.296	8.870	12.470	17.568	22.690	27.871
61	8.898	12.457	17.222	22.560	27.084	0.858	0.817	3.313	8.894	12.504	17.616	22.754	27.952
62	8.905	12.438	17.307	22.150	26.699	0.829	0.811	3.375	8.898	12.512	17.636	22.793	28.014
63	8.906	12.456	17.412	22.191	26.776	0.839	0.813	3.362	8.911	12.526	17.647	22.797	28.010
64	8.915	12.478	17.398	22.242	27.266	0.842	0.814	3.365	8.921	12.531	17.644	22.784	27.984
65	8.928	12.442	17.233	22.108	27.480	0.873	0.823	3.318	8.913	12.499	17.566	22.647	27.779

Table S7. *continued*

	Empirical Distribution					Generalized Gamma distribution							
	Quantiles					Parameter			Quantiles				
m	95%	99%	99.9%	99.99%	99.999%	a	b	κ	95%	99%	99.9%	99.99%	99.999%
66	8.950	12.491	17.376	22.609	27.226	0.828	0.811	3.407	8.947	12.563	17.685	22.837	28.051
67	8.973	12.527	17.480	22.455	26.480	0.829	0.811	3.408	8.965	12.589	17.723	22.886	28.112
68	8.969	12.503	17.400	22.237	27.391	0.855	0.818	3.364	8.970	12.583	17.691	22.821	28.005
69	8.986	12.509	17.318	21.625	26.192	0.865	0.821	3.356	8.982	12.589	17.683	22.794	27.956
70	8.991	12.558	17.440	22.065	25.497	0.852	0.818	3.386	8.986	12.595	17.695	22.815	27.989
71	9.001	12.550	17.505	22.289	26.678	0.821	0.811	3.454	8.999	12.617	17.738	22.884	28.091
72	9.002	12.571	17.516	22.157	26.006	0.823	0.811	3.453	9.009	12.630	17.755	22.907	28.118
73	9.015	12.561	17.559	22.090	27.085	0.817	0.810	3.470	9.014	12.635	17.761	22.914	28.127
74	9.047	12.587	17.628	22.676	27.179	0.839	0.814	3.428	9.042	12.671	17.803	22.957	28.168
75	9.062	12.613	17.523	22.145	26.318	0.824	0.812	3.466	9.041	12.667	17.794	22.946	28.156
76	9.055	12.623	17.476	22.332	27.111	0.861	0.821	3.406	9.055	12.666	17.763	22.872	28.029
77	9.057	12.598	17.545	22.090	26.436	0.857	0.820	3.417	9.049	12.655	17.744	22.845	27.994
78	9.063	12.636	17.534	22.516	27.755	0.844	0.817	3.445	9.063	12.678	17.784	22.904	28.077
79	9.101	12.712	17.670	22.545	27.887	0.777	0.800	3.583	9.102	12.759	17.945	23.167	28.459
80	9.093	12.673	17.562	22.489	26.438	0.824	0.812	3.499	9.097	12.725	17.853	23.000	28.202
81	9.099	12.682	17.572	22.357	27.024	0.822	0.812	3.510	9.102	12.727	17.847	22.987	28.180
82	9.112	12.643	17.519	22.464	26.961	0.831	0.814	3.490	9.106	12.732	17.854	22.992	28.184
83	9.118	12.689	17.715	22.702	26.920	0.817	0.811	3.525	9.109	12.736	17.858	23.000	28.197
84	9.123	12.697	17.686	22.274	26.626	0.816	0.811	3.536	9.125	12.755	17.882	23.029	28.230
85	9.140	12.700	17.713	22.833	27.161	0.818	0.811	3.528	9.136	12.773	17.911	23.069	28.282
86	9.141	12.701	17.632	22.441	26.058	0.843	0.818	3.488	9.132	12.750	17.852	22.964	28.124
87	9.155	12.720	17.583	22.255	26.693	0.839	0.817	3.502	9.151	12.777	17.892	23.018	28.193
88	9.153	12.776	17.679	22.539	26.802	0.793	0.805	3.595	9.157	12.801	17.955	23.132	28.370
89	9.168	12.715	17.586	22.127	26.330	0.819	0.813	3.553	9.157	12.782	17.897	23.027	28.209
90	9.168	12.759	17.674	22.447	26.591	0.813	0.811	3.569	9.169	12.800	17.926	23.067	28.262
91	9.209	12.784	17.659	22.607	27.526	0.798	0.807	3.604	9.199	12.848	18.006	23.184	28.421
92	9.198	12.803	17.710	22.969	26.932	0.797	0.807	3.612	9.196	12.841	17.990	23.159	28.386
93	9.192	12.754	17.767	22.670	27.243	0.814	0.811	3.582	9.195	12.828	17.955	23.096	28.289
94	9.204	12.744	17.679	22.513	26.494	0.805	0.810	3.608	9.197	12.828	17.950	23.088	28.278
95	9.215	12.809	17.747	22.383	26.542	0.787	0.805	3.652	9.212	12.852	17.993	23.154	28.372

Table S7. *continued*

	Empirical Distribution					Generalized Gamma distribution							
	Quantiles					Parameter			Quantiles				
m	95%	99%	99.9%	99.99%	99.999%	a	b	κ	95%	99%	99.9%	99.99%	99.999%
96	9.228	12.832	17.797	22.585	27.306	0.804	0.809	3.616	9.229	12.873	18.016	23.174	28.386
97	9.256	12.834	17.699	22.500	27.553	0.803	0.808	3.621	9.246	12.899	18.056	23.229	28.457
98	9.234	12.829	17.744	22.332	25.907	0.835	0.817	3.562	9.240	12.872	17.988	23.109	28.275
99	9.259	12.839	17.860	22.792	27.995	0.787	0.805	3.666	9.257	12.910	18.069	23.247	28.481
100	9.269	12.849	17.773	22.351	26.986	0.773	0.802	3.700	9.251	12.902	18.060	23.240	28.479
101	9.267	12.887	18.017	23.074	27.444	0.767	0.800	3.711	9.274	12.941	18.126	23.336	28.609
102	9.258	12.890	17.805	22.564	27.150	0.788	0.806	3.669	9.258	12.906	18.056	23.223	28.444
103	9.277	12.863	17.777	22.671	26.588	0.786	0.806	3.684	9.271	12.918	18.065	23.228	28.446
104	9.295	12.856	17.626	22.655	27.811	0.815	0.813	3.627	9.277	12.914	18.039	23.172	28.352
105	9.285	12.849	17.732	22.529	26.875	0.814	0.813	3.635	9.275	12.908	18.025	23.148	28.318
106	9.309	12.880	17.870	22.715	28.572	0.779	0.804	3.714	9.297	12.950	18.105	23.278	28.505
107	9.309	12.897	17.876	22.780	27.052	0.777	0.803	3.717	9.301	12.957	18.119	23.299	28.534
108	9.317	12.907	17.851	22.835	27.530	0.794	0.808	3.692	9.306	12.950	18.086	23.233	28.430
109	9.316	12.895	17.858	22.714	27.181	0.803	0.810	3.674	9.317	12.964	18.101	23.247	28.442
110	9.301	12.901	17.790	22.514	26.855	0.791	0.808	3.704	9.306	12.946	18.076	23.216	28.406
111	9.336	12.912	17.884	22.840	27.528	0.796	0.809	3.696	9.327	12.975	18.114	23.264	28.463
112	9.331	12.926	17.964	22.799	27.529	0.804	0.811	3.686	9.333	12.975	18.104	23.238	28.419
113	9.338	12.933	17.816	22.719	27.293	0.794	0.808	3.706	9.338	12.988	18.131	23.284	28.487
114	9.332	12.929	17.947	23.034	27.860	0.786	0.807	3.729	9.339	12.987	18.127	23.278	28.478
115	9.365	13.000	17.978	22.657	27.116	0.782	0.806	3.746	9.360	13.015	18.166	23.329	28.543
116	9.361	12.963	17.931	22.822	27.318	0.809	0.812	3.685	9.355	13.002	18.134	23.271	28.453
117	9.370	12.956	17.892	22.612	28.129	0.768	0.802	3.779	9.367	13.027	18.188	23.365	28.595
118	9.380	12.934	17.945	22.686	27.267	0.783	0.806	3.751	9.370	13.025	18.174	23.334	28.543
119	9.379	12.993	18.048	23.483	28.257	0.778	0.805	3.760	9.380	13.042	18.205	23.381	28.608
120	9.386	12.954	17.952	22.677	27.270	0.782	0.806	3.761	9.384	13.038	18.186	23.343	28.550
121	9.385	12.979	18.022	22.919	26.774	0.773	0.804	3.781	9.385	13.042	18.196	23.361	28.577
122	9.402	13.010	17.900	22.801	26.981	0.786	0.807	3.754	9.392	13.047	18.195	23.351	28.556
123	9.396	13.011	17.979	22.742	27.647	0.807	0.813	3.718	9.396	13.040	18.165	23.290	28.457
124	9.407	13.007	17.932	23.072	27.739	0.780	0.806	3.779	9.399	13.053	18.199	23.352	28.554
125	9.415	13.005	17.974	22.607	28.488	0.792	0.809	3.755	9.413	13.069	18.215	23.365	28.562

Table S7. *continued*

	Empirical Distribution					Generalized Gamma distribution							
	Quantiles					Parameter			Quantiles				
m	95%	99%	99.9%	99.99%	99.999%	a	b	κ	95%	99%	99.9%	99.99%	99.999%
126	9.397	12.983	17.983	22.944	27.641	0.792	0.809	3.756	9.401	13.048	18.180	23.315	28.494
127	9.417	12.995	17.893	22.667	28.334	0.786	0.808	3.775	9.407	13.055	18.187	23.325	28.507
128	9.434	13.049	17.969	22.896	27.041	0.772	0.804	3.807	9.430	13.093	18.254	23.424	28.644
129	9.439	13.027	17.958	22.765	27.869	0.794	0.810	3.766	9.436	13.090	18.230	23.372	28.557
130	9.454	13.052	18.084	22.891	27.045	0.724	0.792	3.929	9.448	13.131	18.332	23.555	28.839
131	9.440	13.039	17.934	22.491	27.034	0.765	0.803	3.840	9.433	13.088	18.234	23.389	28.593
132	9.456	13.071	18.075	22.428	27.551	0.773	0.805	3.825	9.454	13.114	18.266	23.425	28.632
133	9.456	13.082	18.089	22.903	28.062	0.748	0.799	3.885	9.456	13.124	18.294	23.476	28.713
134	9.465	13.079	18.185	23.013	27.779	0.777	0.806	3.818	9.468	13.133	18.293	23.457	28.670
135	9.464	13.062	17.930	22.912	27.366	0.767	0.804	3.843	9.457	13.118	18.273	23.434	28.645
136	9.472	13.095	17.942	23.058	29.643	0.781	0.807	3.820	9.466	13.121	18.261	23.404	28.591
137	9.463	13.029	17.980	22.759	28.874	0.780	0.808	3.823	9.452	13.097	18.221	23.347	28.517
138	9.491	13.103	18.061	22.578	27.881	0.765	0.803	3.860	9.481	13.146	18.304	23.468	28.681
139	9.480	13.083	18.036	22.975	27.305	0.776	0.806	3.836	9.480	13.139	18.285	23.434	28.628
140	9.491	13.083	18.071	22.872	27.644	0.800	0.812	3.784	9.486	13.140	18.273	23.403	28.573
141	9.488	13.107	18.062	23.020	28.093	0.766	0.804	3.865	9.489	13.151	18.303	23.460	28.664
142	9.510	13.149	18.186	23.360	28.752	0.731	0.795	3.950	9.503	13.184	18.374	23.579	28.841
143	9.507	13.132	18.065	23.055	28.177	0.745	0.799	3.924	9.497	13.163	18.326	23.497	28.720
144	9.500	13.076	18.071	22.767	27.525	0.814	0.816	3.770	9.504	13.151	18.267	23.376	28.519
145	9.518	13.109	17.946	22.558	26.788	0.792	0.811	3.822	9.503	13.153	18.279	23.402	28.564
146	9.514	13.151	18.143	23.088	27.444	0.757	0.802	3.903	9.517	13.185	18.345	23.512	28.726
147	9.516	13.110	18.074	22.894	27.437	0.806	0.814	3.793	9.516	13.166	18.289	23.404	28.555
148	9.544	13.171	18.239	23.161	28.040	0.731	0.795	3.968	9.547	13.236	18.435	23.647	28.915
149	9.532	13.155	18.101	23.119	26.966	0.761	0.803	3.902	9.526	13.190	18.344	23.501	28.704
150	9.530	13.141	18.106	23.130	27.603	0.762	0.804	3.904	9.525	13.185	18.331	23.479	28.673
151	9.542	13.155	18.150	23.160	27.713	0.768	0.805	3.889	9.539	13.205	18.357	23.511	28.709
152	9.571	13.167	18.097	22.743	26.880	0.767	0.804	3.895	9.554	13.226	18.389	23.553	28.763
153	9.554	13.160	18.104	22.866	27.449	0.764	0.804	3.905	9.548	13.215	18.370	23.526	28.727
154	9.564	13.202	18.264	22.833	27.511	0.734	0.796	3.974	9.567	13.257	18.455	23.665	28.929
155	9.586	13.186	18.149	22.928	28.013	0.765	0.804	3.911	9.569	13.242	18.404	23.567	28.775

Table S7. *continued*

	Empirical Distribution					Generalized Gamma distribution							
	Quantiles					Parameter			Quantiles				
m	95%	99%	99.9%	99.99%	99.999%	a	b	κ	95%	99%	99.9%	99.99%	99.999%
156	9.575	13.180	18.245	22.993	28.266	0.783	0.809	3.874	9.572	13.238	18.384	23.526	28.707
157	9.565	13.134	18.183	23.288	28.018	0.768	0.806	3.906	9.558	13.220	18.365	23.509	28.694
158	9.573	13.229	18.280	23.255	27.126	0.736	0.798	3.991	9.573	13.248	18.419	23.597	28.825
159	9.596	13.232	18.205	22.847	27.924	0.758	0.802	3.931	9.595	13.281	18.465	23.653	28.887
160	9.592	13.223	18.345	23.300	28.031	0.731	0.796	4.000	9.599	13.291	18.490	23.698	28.959
161	9.598	13.236	18.150	23.736	29.221	0.751	0.801	3.955	9.593	13.274	18.451	23.632	28.860
162	9.586	13.170	18.183	22.954	27.766	0.770	0.806	3.918	9.587	13.253	18.400	23.545	28.730
163	9.603	13.214	18.164	22.867	27.917	0.752	0.801	3.962	9.601	13.278	18.447	23.619	28.836
164	9.606	13.202	18.215	23.109	27.882	0.765	0.805	3.931	9.592	13.260	18.413	23.564	28.758
165	9.595	13.227	18.269	23.012	27.844	0.743	0.800	3.986	9.600	13.277	18.446	23.619	28.839
166	9.610	13.203	18.296	23.157	27.872	0.781	0.809	3.903	9.607	13.272	18.415	23.551	28.725
167	9.608	13.202	18.316	23.355	27.856	0.760	0.804	3.955	9.596	13.259	18.403	23.544	28.727
168	9.624	13.272	18.319	22.736	27.587	0.745	0.800	3.993	9.626	13.309	18.485	23.665	28.891
169	9.622	13.237	18.241	23.516	29.222	0.732	0.797	4.025	9.618	13.301	18.481	23.667	28.902
170	9.636	13.242	18.175	22.847	27.411	0.771	0.806	3.935	9.629	13.302	18.456	23.605	28.794
171	9.618	13.276	18.248	22.919	27.915	0.756	0.803	3.971	9.624	13.299	18.460	23.621	28.824
172	9.653	13.256	18.241	23.031	27.890	0.755	0.802	3.970	9.639	13.324	18.501	23.678	28.900
173	9.627	13.235	18.226	22.889	27.090	0.738	0.799	4.020	9.618	13.290	18.449	23.611	28.818
174	9.657	13.271	18.275	23.370	28.637	0.756	0.803	3.980	9.643	13.319	18.480	23.639	28.840
175	9.641	13.265	18.216	23.070	27.665	0.761	0.805	3.973	9.639	13.308	18.459	23.604	28.790
176	9.650	13.286	18.215	23.136	28.683	0.754	0.803	3.990	9.647	13.322	18.484	23.642	28.843
177	9.666	13.272	18.204	23.135	27.445	0.776	0.808	3.937	9.650	13.321	18.470	23.612	28.790
178	9.651	13.234	18.232	23.065	27.454	0.751	0.803	4.008	9.637	13.300	18.440	23.577	28.754
179	9.662	13.309	18.200	23.152	28.708	0.720	0.794	4.083	9.661	13.349	18.535	23.727	28.968
180	9.668	13.253	18.093	22.654	27.152	0.749	0.802	4.014	9.653	13.326	18.481	23.634	28.829
181	9.672	13.280	18.414	23.604	28.464	0.726	0.796	4.070	9.670	13.359	18.545	23.736	28.974
182	9.679	13.321	18.283	23.305	28.560	0.736	0.798	4.045	9.675	13.363	18.544	23.727	28.956
183	9.677	13.275	18.295	23.656	28.416	0.769	0.807	3.970	9.670	13.342	18.490	23.631	28.808
184	9.673	13.308	18.334	23.341	27.568	0.752	0.803	4.011	9.677	13.356	18.520	23.681	28.882
185	9.669	13.293	18.205	23.461	29.197	0.764	0.806	3.990	9.668	13.333	18.474	23.606	28.774
186	9.689	13.311	18.174	23.186	28.805	0.754	0.803	4.014	9.683	13.360	18.519	23.673	28.868
187	9.680	13.331	18.243	23.328	28.141	0.743	0.801	4.042	9.676	13.351	18.511	23.668	28.867
188	9.692	13.306	18.251	23.279	27.157	0.743	0.801	4.045	9.681	13.355	18.513	23.666	28.861
189	9.707	13.346	18.307	23.146	27.097	0.733	0.798	4.072	9.694	13.378	18.551	23.723	28.940
190	9.692	13.307	18.248	23.037	28.796	0.763	0.806	4.003	9.686	13.353	18.493	23.624	28.791
191	9.706	13.358	18.323	22.968	26.656	0.725	0.796	4.096	9.707	13.398	18.583	23.770	29.004
192	9.691	13.314	18.302	23.165	28.372	0.758	0.805	4.017	9.687	13.354	18.496	23.630	28.801
193	9.701	13.348	18.215	22.919	28.845	0.744	0.801	4.051	9.699	13.378	18.539	23.696	28.894
194	9.701	13.319	18.237	22.697	26.703	0.759	0.805	4.020	9.694	13.361	18.501	23.633	28.800
195	9.742	13.387	18.351	23.387	28.453	0.723	0.795	4.105	9.731	13.431	18.632	23.835	29.086
196	9.726	13.364	18.332	22.648	26.108	0.742	0.801	4.061	9.719	13.404	18.576	23.744	28.953
197	9.719	13.344	18.294	23.069	28.320	0.750	0.803	4.051	9.714	13.387	18.537	23.678	28.858
198	9.721	13.383	18.293	23.401	28.433	0.742	0.801	4.062	9.725	13.413	18.589	23.761	28.975
199	9.746	13.362	18.373	23.280	27.971	0.728	0.798	4.106	9.732	13.420	18.599	23.776	28.997
200	9.727	13.368	18.452	23.478	27.455	0.725	0.797	4.111	9.727	13.417	18.597	23.777	29.002

Figure S9. The histogram of the proposed test statistic after the log-transformation ($-2\log(\text{GATE})$) for $k \in \{1, 2\}$ when the dimension $m = 20$ based on 1,000,000 replicates. The red curve is the density function of the generalized Gamma distribution with the scale parameter a, the shape parameter b and κ whose estimators are presented in the legend.

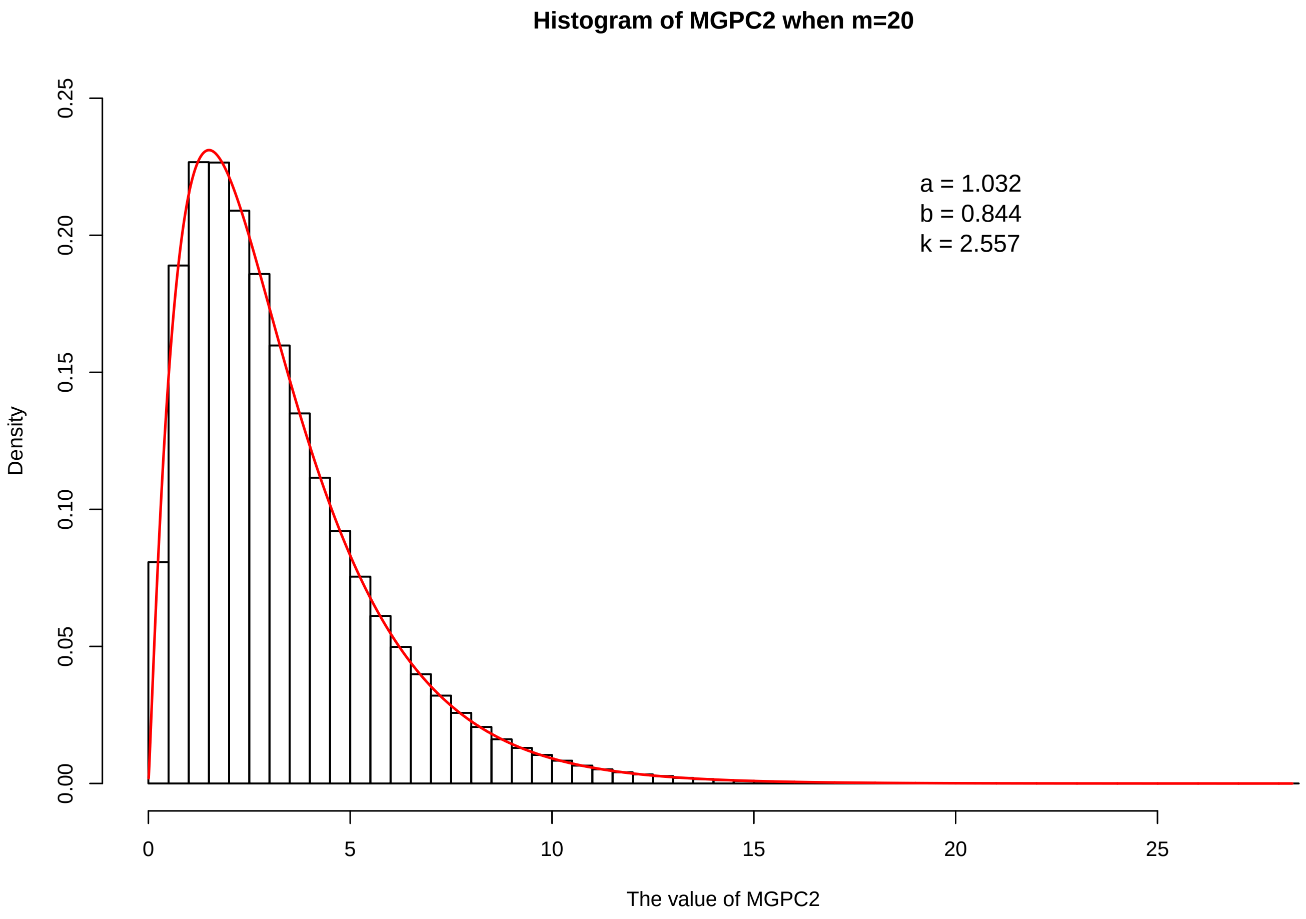

Figure S10. The histogram of the proposed test statistic after the log-transformation $(-2\log(\text{GATE}))$ for $k \in \{1, 2\}$ when the dimension $m = 100$ based on 1,000,000 replicates. The red curve is the density function of the generalized Gamma distribution with the scale parameter a, the shape parameter b and κ. whose estimators are presented in the legend.

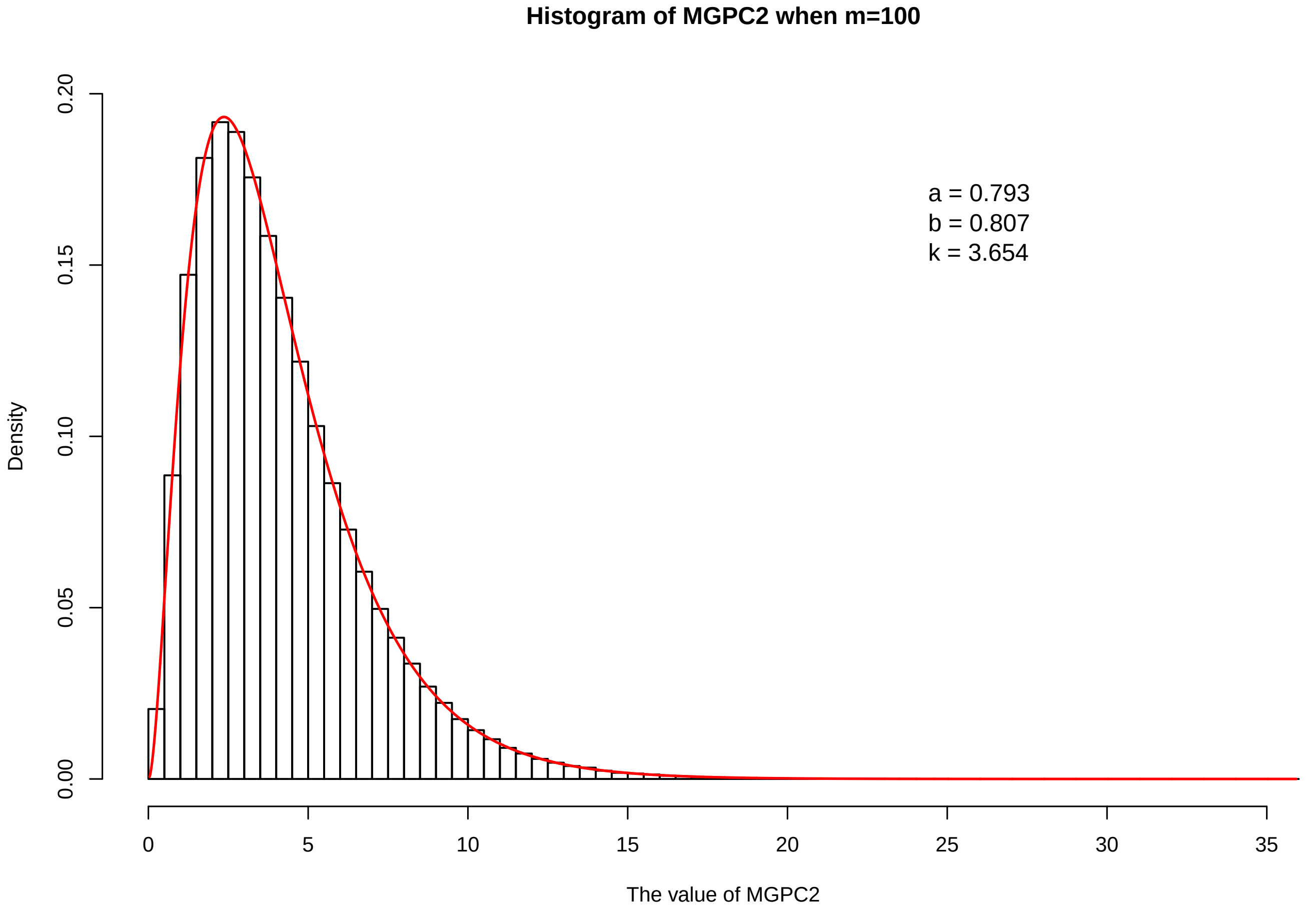

9. The analyzed mice phenotype information.

Table S8. 52 mice phenotypes used in the real applications. The first column is the phenotype name and the corresponding categories that they are belong to are in column 2.

Phenotype.name	category
Adrenal Weight	AdrenalWeight
Serum Chloride	Biochemistry
Fear Conditioning Time freezing to context	Context
Fear Conditioning Fecal boli after cue	Cue
Fear Conditioning Time freezing during cue	Cue
Fear Conditioning Time freezing after cue	Cue
EPM Closed Arm Entries	EPM
EPM Open Arm Distance	EPM
EPM Open Arm Entries	EPM
EPM Open Arm Time	EPM
FPS Change in startle after training	FPS
FPS Startle response	FPS
IPGTT Glucose 0 mins	Glucose
Haem Haemoglobin	Haematology
Haem Mean corpuscular haemglobin	Haematology
Haem Mean cellular Hb conc	Haematology
Haem Mean cellular volume	Haematology
Haem Neutrophils	Haematology
Haem Platelets	Haematology
Haem Red blood cell count	Haematology
Haem Red cell distribution width	Haematology
Haem White blood cell count	Haematology
Haem Haematocrit	Haematology
Imm CD4+/CD8+	Immunology
Imm CD4Intensity	Immunology
Imm CD8Intensity	Immunology
Imm %B220+	Immunology
Imm %CD3+	Immunology
Imm %CD4+	Immunology
Imm %CD4+/CD3+	Immunology
Imm %CD8+	Immunology
Imm %NK cells (inferred)	Immunology
Body Length	Obesity
HomeCage Total beam breaks (First five minutes)	PAS
HomeCage Total beam breaks (30 minutes)	PAS
HomeCage Fine Movement	PAS
OFT Activity and defecation	EMO
OFT Faecal Boli	OFT
OFT Center Time	OFT
OFT Total Activity	OFT
Pleth Respiratory rate (baseline)	Plethysmograph
Pleth Enhanced pause (baseline)	Plethysmograph
Pleth Inspiratory time (baseline)	Plethysmograph
Pellets Burrowed	Burrowing
Weight 10 weeks	Weight
Weight 7 weeks	Weight
Weight 8 weeks	Weight
Weight 6 weeks	Weight
Ear hole area after ear-punch at 6 weeks	EarPunch
EPM Open Arm Latency	EPM
FN Latency to eat novel food	FN
OFT Latency	OFT
